# Supplementary material for: Antimalarial mass drug administration in large populations and the evolution of drug resistance
Source: PLOS Glob Public Health. 2023 Jul 26;3(7):e0002200. doi: 10.1371/journal.pgph.0002200 (PMC10370688; doi:10.1371/journal.pgph.0002200)
Supplement: S1 Text — This PDF file contains information concerning the model parameterization, calibration, validation, and additional sensitivity analyses. (PDF) [file pgph.0002200.s001.pdf]

Supplementary Appendix 1 to "Antimalarial mass drug administration in large populations and the evolution of drug resistance" by Nguyen, Tran, Parker, et al

1 Mechanisms, Parameterizations, and Calibration of Individual-Based Malaria Simulation

Full model details are located in the supplement (open-access) to the original Nguyen et al paper [1] from 2015 which can be downloaded here: https://www.thelancet.com/cms/10.1016/S2214-109X(15)00162-X/attachment/dc5c729e-02cc-4d4e-9d61-35ad6894299c/mmc1.pdf This supplement details 14 separate mechanisms in malaria infection and epidemiology that need to parameterized/calibrated when building a mathematical model of malaria transmission. Thirty data sets, described in the supplement, were used for this calibration. A summary of all major model mechanisms is presented below.

Briefly, the model is a stochastic individual-based simulation coded in C++ and run with a daily time step. In the present analysis 40,000 to 300,000 individuals are instantiated in the model as the population of focus that will undergo mass drug administration (MDA). Each day, for each *P. falciparum* genotype, a Poisson number of individuals is drawn to be bitten by a mosquito; the mean of this Poisson distribution is based on the force of infection of that genotype 11 days ago, to account for the life cycle of the parasite in the mosquito from gametocyte to sporozoite. And the force of infection, in turn, is determined by the number of individuals infected 11 days ago, weighted by their genotype-specific parasitaemia levels. Individuals that are already harboring a single parasite population can be bitten by an infectious mosquito and will then harbor two parasite populations (resulting in a multi-clonal infection, or MOI = 2). Additionally, the force of infection for each genotype is adjusted (using a traditional population-genetic recombination table) for the probability of recombination during mosquito bites that occur on individuals with multi-clonal infections.

Important and central model behaviors described below. Model behaviors that have not changed since 2015 are reviewed briefly.

1.1 Parasite Density

Asexual blood stage parasitaemia in the model is tracked for each person and each clonal parasite population (or clone, or genotype) separately. Symptomatic individuals start with an asexual parasite density between 2000 parasites per microliter (/µl) and 200,000/µl. For symptomatic hosts, this number is drawn uniformly on a $\log_{10}$-scale between 3.301 and 5.301. For new bites that progress to asymptomatic or sub-clinical infection, $\log_{10}$-parasite density is drawn from a log-normal distribution with mean 3.0 and standard deviation 0.5, in other words approximately 1000 parasites per microliter. Asymptomatic density will get progressively lower as the immune system works to reduce the overall parasite density. Asymptomatic infections last between 60 and 281 days, depending on the host's initial immune status (see parameterization and data sources in Nguyen et al [1]). In the blood stage, non-wild-type clones experience a cost of resistance in the absence of drug pressure. For a single-resistant clone (i.e. a genotype with one resistant/mutated allele or one CNV), the daily cost of resistance is c_R = 0.0005, which results in a 17% genotype

frequency drop over one year compared to a wild-type parasite population. The daily cost of resistance for a parasite clone with n genetic mechanisms (non-wild-type) conferring resistance is $c_R = 1 - (1 - 0.0005)^n$.

Gametocytaemia is not explicitly modeled, but adults (children) in the first six (four) days of their blood-stage infection are not infectious to mosquitoes due to lack of gametocytes. Because gametocytes are not explicitly modeled, we cannot evaluate gametocytocidal drugs and the differential effects they are supposed to have during the period after symptoms resolve. In general, asexual parasitaemia is an adequate proxy for transmissibility, but this will not be true after treatment with primaquine or tafenoquine (these drugs are not included in the present analysis).

1.2 Immunity

The level of acquired malaria immunity is modeled as non-antigen-specific immunity to falciparum in general, with the current immune level described simply as being between zero (no immune response of memory) and one (maximum possible effect of malaria immunity in adults resulting in protection from clinical symptoms of malaria infection). Immunity reduces probability of symptoms when infected and reduces parasitaemia more quickly when immunity is high. No changes have been made to the immune system model since from the original published simulation [1]. Children acquire immunity more slowly than adults. When not exposed to malaria, immunity wanes substantially after 18 to 24 months (90% immune loss after 2.5 years [2]). The effect of immunity on symptoms progression was validated using field data comparing clinical episodes in 2-year-olds, 10-year-olds, and 17-year-olds in different transmission settings (sections 11 and 12 in supplement to [1]).

1.3 New Locus Structure

To allow for partial resistance and multiple levels of drug resistance due to mutations at different loci, a new locus representation was introduced into the model. This locus-based model includes positions N86Y and Y184F in the *pfmdr1* gene on chromosome 5. Copy number variation (CNV) is allowed for *pfmdr1*, but the alleles on the second copy are restricted to be the same as the alleles on the first copy. Eight configurations (haplotypes) are possible for chromosome 5, and they can be coded or summarized in the following shorthand: `NY--`, `YY--`, `NF--`, `YF--`, `NYNY`, `YYYY`, `NFNF`, `YFYF`. The dashed lines indicate an absence of a second copy of *pfmdr1*. The model does not distinguish between two copies or more than two copies.

On chromosome 7, the K76T alleles are included for the *pfcrt* gene. On chromosome 13, the C580Y alleles are included for the *pfkelch13* gene. And a generic piperaquine-resistant genotype (labelled as "`1`" for absent and "`2`" for present) is included. This phenotype used to be associated with copy number variation in allowed for the *plasmepsin-2,3* genes [3,4] but now is believed to be more closely associated with certain alleles (not K76T) in the *pfcrt* gene [5–10].

This results in 64 total genotypes, where the wild-type can be coded as `KNY--Y1` or `KNY1Y1` if we choose to use the "1" to indicate one copy of *pfmdr1*. Details of how these 64 genotypes map to phenotypes (resistant phenotypes associated with particular treatment efficacies) is in S2 Text.

The chromosomal positions of the loci are important because our model includes recombination (sexual reproduction) at the point of transmission to the mosquito, when a mosquito bites a multi-clonally infected host. The force of infection of an individual infected with multiple clones is the sum of all forces of infection for all possible recombinant offspring that could be produced with the host's parasites. The potential recombinant offspring are listed using a standard recombination table, and the entries in the table (the probabilities of forming a particular recombinant based on two parental genotypes) account for the current parasite densities of clones in an individual's blood. No interrupted mosquito feeding is modeled, so recombination only occurs if there is multi-clonality in the populations.

1.4 Pharmacokinetics and Pharmacodynamics (PK/PD)

The individual-based simulation uses a simple 1-compartment PK model with drug-clearance half-life as the key parameter that determines the exponential waning of drugs in the blood. Since the simulation runs with a 1-day timestep, artemisinin derivatives are cleared from the drug on the same day and are associated with a kill rate of 0.998 per day. Half-life for lumefantrine is 4.5 days, amodiaquine 9.0 days, and piperaquine 28.0 days. Pharmacodynamic properties were calibrated on a per-genotype basis. Parasite killing takes place according to a classic Hill equation ($C^n / (C^n + \mathrm{EC50}^n)$) and the EC50 parameters were calibrated to achieve the genotype-specific 28-day efficacies in presented in S2 Text. For lumefantrine $n = 20$, for amodiaquine $n = 19$, and for piperaquine $n = 15$. This classic 1-compartment PK/PD framework is the same as the one used in the original 2015 paper; only the parametrizations in S2 Text are new.

1.4.1 Pharmacokinetics and Pharmacodynamics of OZ439 and Ferroquine

These two drugs were used in an experimental set of simulations (Fig C to Fig E) where we examined the benefits of substituting out artemether-lumefantrine (AL) as first-line therapy after the MDA and replacing it with a non-artemisinin combination therapy of OZ439 and ferroquine (FQ). Half-life of OZ439 was assumed to be 2.5 days [11] and half-life of FQ was taken as 10 days [12]. In McCarthy et al [13], OZ439 plasma concentration half-life was reported as 260h (geometric mean) for a 500mg dose, while in the 200mg dose group the half-life was 51h. Hence, we chose 2.5 days (60h) as the plausible value for OZ439 half-life.

The 48-hour parasite reduction ratio (PRR_{48}) of OZ439 was estimated to be 10176 (95% CI 5757 - 17986) for a 500mg dose and 165 (95% CI 124 -222) for 200 mg doses [13]. In this case, the PRR_{24} estimate will be 100.86 (95% CI 75.87 – 134.11) for a 500mg dose; i.e. the fractional parasite killing per day will be in the range 0.9700 to 0.9925. In Phyo et al [11], the killing rate per hour was estimated to be 0.13 and 0.17, for 400mg and 800mg doses, respectively; which yields a range of parasite killing per day between 0.9646 and 0.9886. In the simulation, we set the maximum fraction of parasites that can be killed per day ($p_{\max}$) to be 0.9990 for a host with a very high (40% above average) OZ439 drug concentration. For an average drug concentration (C_0=1.0) with EC_{50} = 0.75, the Hill factor $C^n / (C^n + \mathrm{EC50}^n)$ results in a killing rate of 0.9467, just below the indicated range above.

For FQ, the PRR_{48} was reported as 163 (95 % CI 141–188) which is equivalent to PRR_{24} of 12.77 (95% CI 11.87 – 13.71) [3]. Daily fractional parasite killing is estimated to be between 0.9157 and 0.9271. In the simulation we choose

the maximum fraction of parasites that can be killed per day to be $p_{\max}$ = 0.99 for the host which results in > 0.9 killing rates for high drug concentrations.

The efficacy of the combination OZ439-FQ was calculated by running five simulations on 10,000 patients each. Each patient had a per-μl parasitaemia drawn from a log-uniform distribution between 2000 and 200,000. 28-day efficacies ranged from 95.5% to 95.9% in the five simulated trials.

Summary of the PK/PD parameters used in the simulation for OZ439 and FQ:

	OZ439	FQ
Half-life ($t_{0.5}$)	2.5 days (Phyo et al., 2016)	10 days (McCarthy et.al., 2016)
The maximum fraction of parasites that can be killed per day (p_{max})	0.999	0.99
Slope of the concentration-effect curve (n)	10	5
The drug concentration at which the parasite killing is 50% of $p_{\max}$ (EC_{50})	0.75	0.75

1.5 Mutation Rate Calibration

The model's mutation rate – as in all large-scale population-level models of malaria – is a combination of the parasite's mutation rate and the probability that a newly arrived mutant will fix within-host. This is the reason that most models describe this process as a person being treated with a drug and *their parasites mutating* (i.e. all of them) from drug-sensitive to drug-resistant genotypes. The true process requires a *de novo* mutation to occur in a single parasite, and then a selection process to take place (viability selection, due to the high killing rates of drugs) that results in a within-host falciparum population that, 14 generations or 28 days later, is majority drug resistant. Because neither the within-host fixation rates nor the allele-specific mutation rates are known with enough accuracy, this process was calibrated using a longer-term population-level outcome.

Three academic groups working in individual-based malaria modeling (Oxford, Imperial College, Penn State) participated in a calibration exercise to align their models so that mutants would appear at approximately the same rate, making model runs comparable to each other across the three groups [14]. The *kelch13* locus was chosen as the critical locus to align on as artemisinin-resistance emergence will be a topic of interest and analysis for years to come. The first *kelch13* artemisinin-tolerant mutants arose sometime in the 1990s in western Cambodia, a region with hundreds of thousands of individuals. The frequency of the most successful of these mutants (C580Y) probably rose to 0.01 allele frequency within 3-10 years of its emergence. This is a reasonable assumption given that the mutant's frequency was around 0.40 in 2001-2002, but there are no data or samples pre-2001. To mimic this process, the three groups calibrated their models on a population of 100,000 individuals, with PfPR=10%, and 40% DHA-PPQ use in the population. All groups adjusted their individual (and differently implemented) mutation-rate parameters so that

580Y alleles would reach 0.01 allele frequency in 7.0 years exactly. This give us a mutation process that is within one order of magnitude of the true process. The MDA policy itself is not much affected by the value of this mutation parameter. In Fig 2 and Fig 3 of the main paper, there are hundreds or thousands of parasite-positive individuals present during the implementation of the MDA, a number much too small to produce a meaningful number of *de novo* mutants, even with a mutation rate one or two orders of magnitude higher.

1.6 Public and Private Market

In the new model, individuals can seek and receive malaria treatment either in the public sector or in the private market. In the baseline scenario set-up, the fraction of public market use starts at 5% (during the 14-year model burn-in) and increases linearly to 80% in year 20, which means that the fraction of private market use will wane from 95% to 20% from over a 34-year period (something like 2010 to 2044, if the MDA were to take place in 2024). This means that when the MDA takes place, approximately 36% of malaria treatment seeking occurs in the public sector (recommended first-line therapy used) while 64% occurs in private markets or pharmacies (off-policy purchases). In the baseline set-up presented here, artemether-lumefantrine (AL) is the recommended first-line therapy in the public sector, while private-market drug purchases include sulfadoxine-pyrimethamine (30%), amodiaquine monotherapy (30%), chloroquine (30%), and AL (10%). As there are no loci encoding SP resistance in the model, SP efficacy is assumed to be 40% (Fig 2 and Fig 3), 60% (Fig I and Fig K), or 80% (Fig J and Fig L).

1.7 Importation

The new model allows for importation of new genotypes into the population. In the baseline setting for importation scenarios, a new parasite importation follows a Poisson process with a new import occurring on average every 10 days (unless specified otherwise); the imported parasite has a 50% probability of carrying the 580Y allele and a 50% probability of carrying the piperaquine-resistant genotype, independently. All 64 genotypes have an equal 1/64 probability of being imported into the population.

1.8 Mass Drug Administration

The new model contains a mechanism for mass drug administration (MDA). Not all individuals in a population will participate in each round of the MDA. Participation rates vary depending on the communication strategy prior to the MDA, the size of the population, the number of study staff, and the occupations and livelihoods of the residents living in the area. Participation rates that are considered low are in the 60% range while a high participation rate would be around 85%. Children and elderly individuals are likely to have higher participation rates. Individuals that miss one round of MDA are more likely to miss another round, due to their occupation, travel patterns, hesitancy, or some other reason. Therefore, the missingness across rounds is not random across individuals in the population. To model the non-random missingness in an MDA round, each individual in the model is given a probability of MDA participation. These probabilities are drawn from a beta distribution with mean 0.85 (children and elderly) or 0.75 (working age adults, and older children, ages 10-40) for each round. The standard deviation of the beta-distribution is 0.3 to capture the variation in participation and non-participation across individuals in the population, as parameterized from field trials in Myanmar [15,16].

Between one and four rounds of MDA take place in the scenarios modeled here. In the simulation, the duration of each round of MDAs is 14 days and rounds are 5 weeks apart. Individual who participate will be assigned a particular day (at random, uniformly) during the 14 days of the MDA period to receive the first day of their 3-day DHA-PPQ course.

1.9 Improved Treatment Coverage (ITC)

During the MDA campaign, the treatment coverage for symptomatic individuals increases from ~50% to ~80% for a few months. We evaluated scenarios where the infrastructure from MDA scale-up was reused to maintain the high treatment coverage, with the highest levels modeled at 80% drug coverage for routine malaria infections. After the MDA, treatment coverage improved linearly over a six-month period before reaching its maximum of 80% (or another ITC value if specified).

References

1. Nguyen TD, Olliaro P, Dondorp AM, Baird JK, Lam HM, Farrar J, et al. Optimum population-level use of artemisinin combination therapies: a modelling study. Lancet Glob Health. 2015;3: e758–e766.

2. Filipe J a N, Riley EM, Drakeley CJ, Sutherland CJ, Ghani AC. Determination of the processes driving the acquisition of immunity to malaria using a mathematical transmission model. PLoS Comput Biol. 2007;3: e255. doi:10.1371/journal.pcbi.0030255

3. Amato R, Pearson RD, Almagro-Garcia J, Amaratunga C, Lim P, Suon S, et al. Origins of the current outbreak of multidrug-resistant malaria in southeast Asia: a retrospective genetic study. Lancet Infect Dis. 2018;18: 337–345. doi:10.1016/S1473-3099(18)30068-9

4. Witkowski B, Duru V, Khim N, Ross LS, Saintpierre B, Beghain J, et al. A surrogate marker of piperaquine-resistant Plasmodium falciparum malaria: a phenotype–genotype association study. Lancet Infect Dis. 2017;17: 174–183. doi:10.1016/S1473-3099(16)30415-7

5. Okombo J, Mok S, Qahash T, Yeo T, Bath J, Orchard LM, et al. Piperaquine-resistant PfCRT mutations differentially impact drug transport, hemoglobin catabolism and parasite physiology in Plasmodium falciparum asexual blood stages. PLOS Pathog. 2022;18: e1010926. doi:10.1371/journal.ppat.1010926

6. Small-Saunders JL, Hagenah LM, Wicht KJ, Dhingra SK, Deni I, Kim J, et al. Evidence for the early emergence of piperaquine-resistant Plasmodium falciparum malaria and modeling strategies to mitigate resistance. PLOS Pathog. 2022;18: e1010278. doi:10.1371/journal.ppat.1010278

7. Wicht KJ, Small-Saunders JL, Hagenah LM, Mok S, Fidock DA. Mutant PfCRT Can Mediate Piperaquine Resistance in African Plasmodium falciparum With Reduced Fitness and Increased Susceptibility to Other Antimalarials. J Infect Dis. 2022;226: 2021–2029. doi:10.1093/infdis/jiac365

8. Agrawal S, Moser KA, Morton L, Cummings MP, Parihar A, Dwivedi A, et al. Association of a Novel Mutation in the Plasmodium falciparum Chloroquine Resistance Transporter With Decreased Piperaquine Sensitivity. J Infect Dis. 2017;216: 468–476. doi:10.1093/infdis/jix334

9. Dhingra SK, Small-Saunders JL, Ménard D, Fidock DA. Plasmodium falciparum resistance to piperaquine driven by PfCRT. Lancet Infect Dis. 2019;19: 1168–1169. doi:10.1016/S1473-3099(19)30543-2

10. Ross LS, Dhingra SK, Mok S, Yeo T, Wicht KJ, Kümpornsin K, et al. Emerging Southeast Asian PfCRT mutations confer Plasmodium falciparum resistance to the first-line antimalarial piperaquine. Nat Commun. 2018;9: 3314. doi:10.1038/s41467-018-05652-0

11. Phyo AP, Jittamala P, Nosten FH, Pukrittayakamee S, Imwong M, White NJ, et al. Antimalarial activity of artefenomel (OZ439), a novel synthetic antimalarial endoperoxide, in patients with Plasmodium falciparum and Plasmodium vivax malaria: an open-label phase 2 trial. Lancet Infect Dis. 2016;16: 61–69. doi:10.1016/S1473-3099(15)00320-5

12. McCarthy JS, Rückle T, Djeriou E, Cantalloube C, Ter-Minassian D, Baker M, et al. A Phase II pilot trial to evaluate safety and efficacy of ferroquine against early Plasmodium falciparum in an induced blood-stage malaria infection study. Malar J. 2016;15: 469. doi:10.1186/s12936-016-1511-3

13. McCarthy JS, Baker M, O'Rourke P, Marquart L, Griffin P, Hooft van Huijsduijnen R, et al. Efficacy of OZ439 (artefenomel) against early Plasmodium falciparum blood-stage malaria infection in healthy volunteers. J Antimicrob Chemother. 2016;71: 2620–2627. doi:10.1093/jac/dkw174

14. Watson OJ, Gao B, Nguyen TD, Tran TN-A, Penny MA, Smith DL, et al. Pre-existing partner-drug resistance facilitates the emergence and spread of artemisinin resistance: a consensus modelling study. Lancet Microbe. 2022. p. 2021.04.08.437876. doi:10.1101/2021.04.08.437876

15. Landier J, Kajeechiwa L, Thwin MM, Parker DM, Chaumeau V, Wiladphaingern J, et al. Safety and effectiveness of mass drug administration to accelerate elimination of artemisinin-resistant falciparum malaria: A pilot trial in four villages of Eastern Myanmar. Wellcome Open Res. 2017;2: 81. doi:10.12688/wellcomeopenres.12240.1

16. Parker DM, Tun STT, White LJ, Kajeechiwa L, Thwin MM, Landier J, et al. Potential herd protection against Plasmodium falciparum infections conferred by mass antimalarial drug administrations. Ferguson NM, Stresman G, editors. eLife. 2019;8: e41023. doi:10.7554/eLife.41023

2 Additional Scenarios

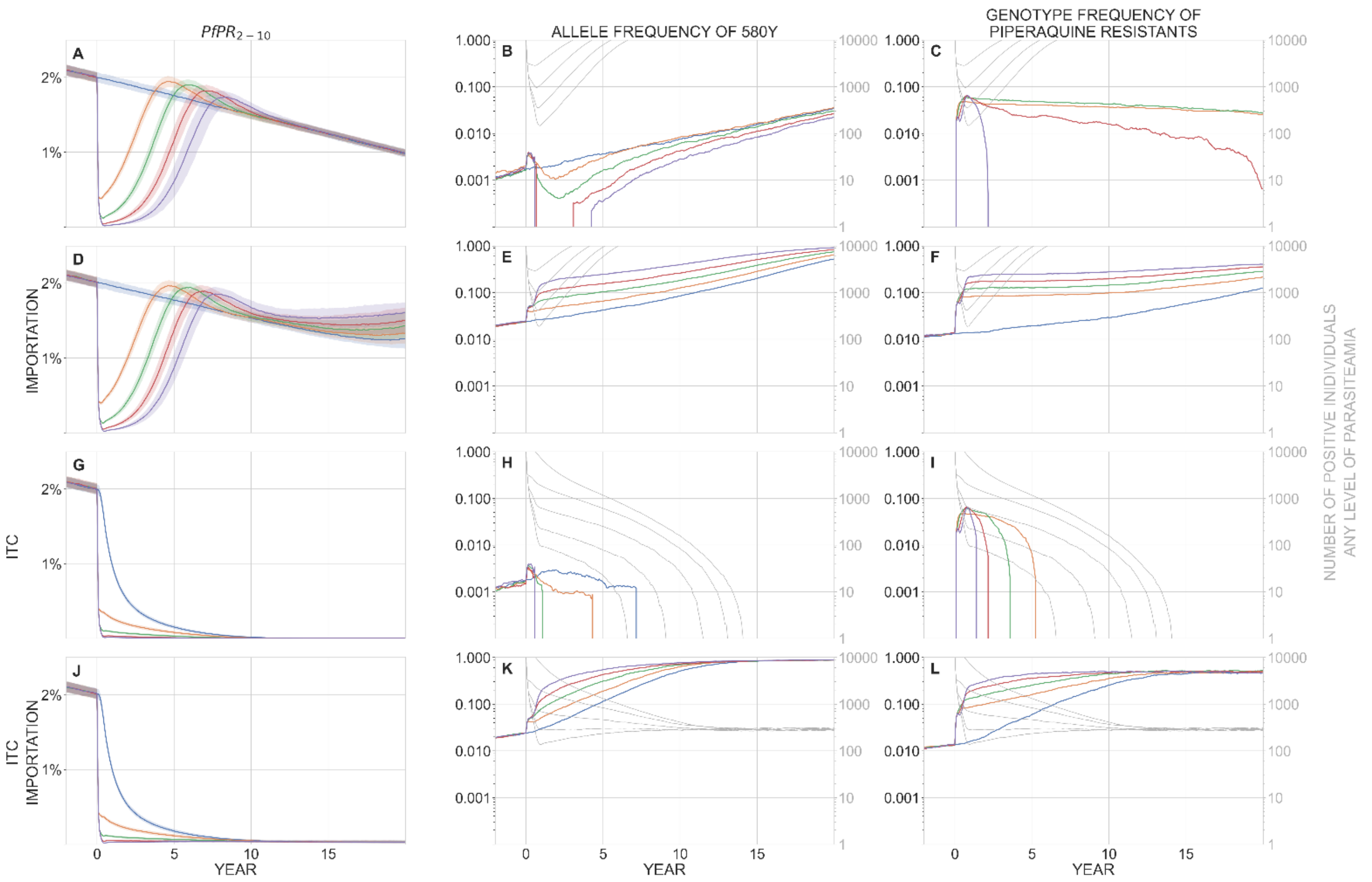

Fig A. In a population of 300,000 individuals, panels show malaria prevalence ($PfPR_{2\text{-}10}$, left), allele frequency of 580Y (middle), and the genotype frequency of piperaquine-resistant parasites (right) over a period of 20 years after MDA has been carried out. In this scenario, baseline $PfPR_{2\text{-}10} = 2\%$. Median trajectories are shown from 1000 simulations (*N*=100 simulations shown in Fig O), and the shaded areas (left column only) show the interquartile range. Simulations are colored by the number of rounds of MDA carried out: blue (0), orange (1), green (2), red (3), purple (4). The top row shows a scenario with no importation of drug-resistant genotypes and no improvement in treatment coverage (ITC) after the MDA is carried out. The second row (panels **D, E, F**) shows a scenario where a new parasite importation occurs every 10 days, on average; the imported parasite has a 50% probability of carrying the 580Y allele and a 50% probability of carrying piperaquine resistance, independently. The third row (panels **G, H, I**) shows a scenario with no importation, but where treatment coverage is increased post-MDA to 80% of the symptomatic patient population. The fourth row (panels **J, K, L**) shows a scenario with both importation and ITC. In the middle and right columns, the light gray lines show the absolute number of infected individuals in the simulation (of any parasitaemia level) and correspond to the right-hand gray tick marks on each panel. Note for example that in panels **K** and **L**, artemisinin-resistant and piperaquine-resistant genotype frequencies are very high, but in a population of only about 300 infected individuals, most of which are imports that occur during the course of the simulation; this region appears to have eliminated malaria but imported parasite-positive individuals can still be found in the region. In all scenarios, the bottleneck period lasts months to years, depending on the number of rounds of MDA carried out. Rapid selection of 580Y can be seen during the bottleneck period when importation is present (panels **E** and **K**). Piperaquine-resistant genotypes are maintained in the population through linkage disequilibrium with 580Y genotypes (panels **F** and **L**, see Fig F). The bottleneck period is risky when importation of 580Y alleles is expected; under these conditions, more rounds of MDA result in worse long-term drug-resistance outcomes.

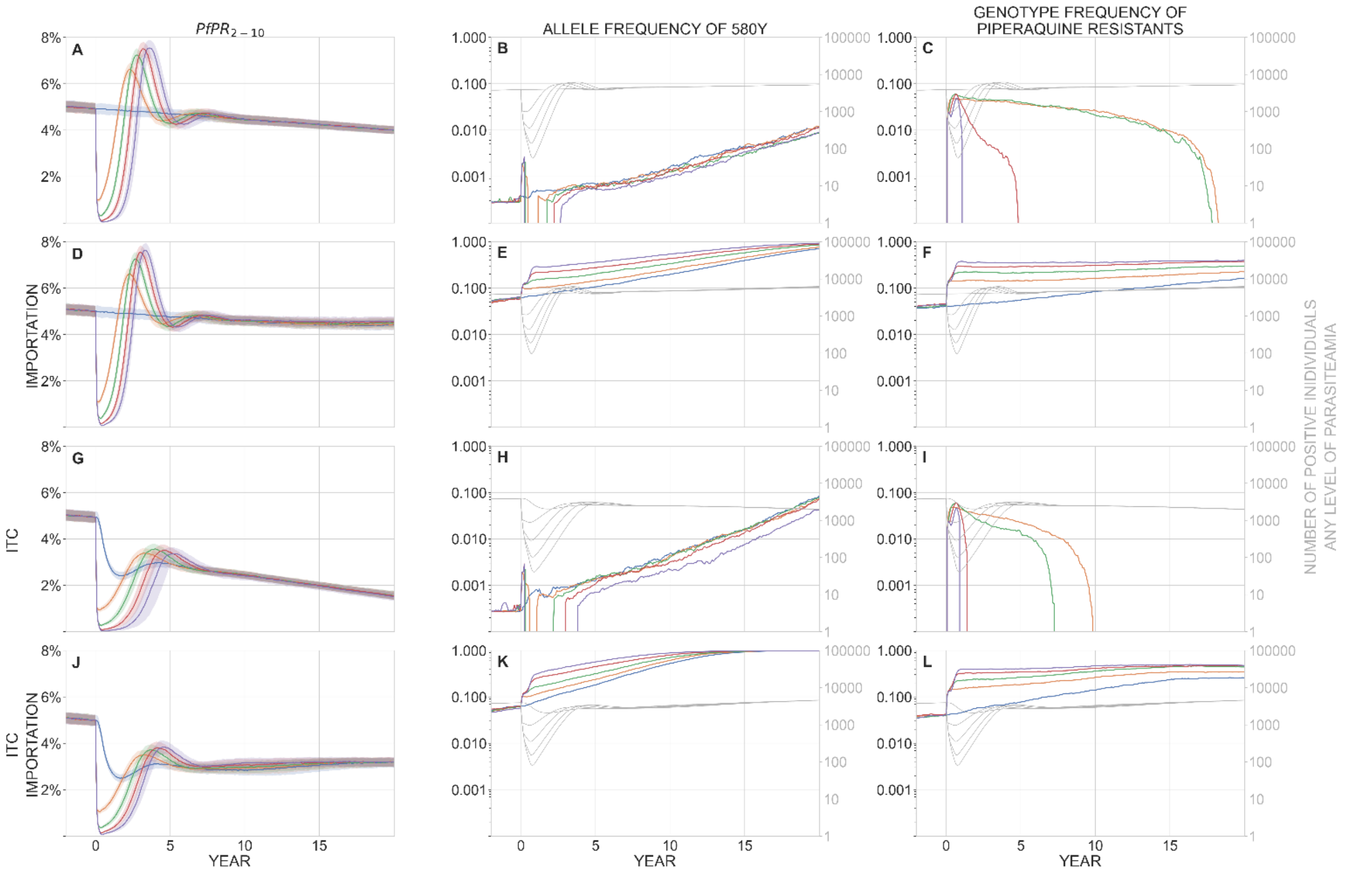

Fig B. In a population of 40,000 individuals, panels show malaria prevalence ($PfPR_{2-10}$, left), allele frequency of 580Y (middle), and the genotype frequency of piperaquine-resistant parasites (right) over a period of 20 years after a mass drug administration has been carried out. In this scenario, baseline $PfPR_{2-10}$ = 5%. Median trajectories are shown from 1000 simulations (N=100 simulations shown in Fig N), and the shaded areas (left column only) show the interquartile range. Simulations are colored by the number of rounds of MDA carried out: blue (0), orange (1), green (2), red (3), purple (4). The top row shows a scenario with no importation of drug-resistant genotypes and no improvement in treatment coverage (ITC) after the MDA is carried out. The second row (panels **D, E, F**) shows a scenario where a new parasite importation occurs every 10 days, on average; the imported parasite has a 50% probability of carrying the 580Y allele and a 50% probability of carrying piperaquine resistance, independently. The third row (panels **G, H, I**) shows a scenario with no importation, but where treatment coverage is increased post-MDA to 80% of the symptomatic patient population. The fourth row (panels **J, K, L**) shows a scenario with both importation and ITC. In the middle and right columns, the light gray lines show the absolute number of infected individuals in the simulation (of any parasitaemia level) and correspond to the right-hand gray tick marks on each panel. In all scenarios, the bottleneck period lasts months to years, depending on the number of rounds of MDA carried out. Selection of 580Y can be seen during the bottleneck period when importation is present (panels **E** and **K**), although this bottleneck effect is weaker in a higher prevalence scenario (this figure) than in a low prevalence scenario (Fig 2E and Fig 2K, main paper). Piperaquine-resistant genotypes are maintained in the population through linkage disequilibrium with 580Y genotypes (panels **C, F, L**, see Fig G). The bottleneck period is risky when importation of 580Y alleles is expected; under these conditions, more rounds of MDA result in worse long-term drug-resistance outcomes.

3 Benefits of removing AL selection pressure post-MDA

Fig C to Fig E show the benefit of replacing AL with an OZ439-FQ combination after the MDA has completed (or just before it starts). Removing selection pressure by AL decreases the risk that artemisinin-resistant genotypes will rise to high genotype frequencies after the MDA-induced bottleneck. OZ439-FQ is used as an example of a non-artemisinin combination therapy that has no cross-resistance with lumefantrine.

Population modeled is 40,000 individuals. Panels show malaria prevalence ($PfPR_{2\text{-}10}$, left), allele frequency of 580Y (middle), and the frequency of piperaquine resistance (right) over a period of 20 years after a mass drug administration has been carried out. In this scenario, baseline $PfPR_{2\text{-}10}$ = 2%. Median trajectories are shown from 100 simulations, and the shaded areas show the interquartile range. Simulations are colored by the number of rounds of MDA carried out: blue (0), orange (1), green (2), red (3), purple (4). In all simulations, a new parasite importation occurs every 10 days, on average; the imported parasite has a 50% probability of carrying the 580Y allele and a 50% probability of carrying piperaquine resistance, independently. In each figure, the top row shows a scenario with no improvement in treatment coverage (ITC) after the MDA is carried out and the bottom row shows a scenario with ITC. In the middle and right columns, the light gray lines show the absolute number of infected individuals in the simulation (of any parasitaemia level) and correspond to the right-hand gray tick marks on each panel. The fixation of 580Y and *plasmepsin-2,3* copy in the **E** and **F** panels was due to the 25% chance of importing double-resistant genotypes (to PPQ and artemisinin) while the number of positive individuals dropped below 100 in the population.

Note that despite high frequencies of 580Y alleles in certain scenarios, these genotypes are not associated with treatment failure since OZ439-FQ is first-line therapy.

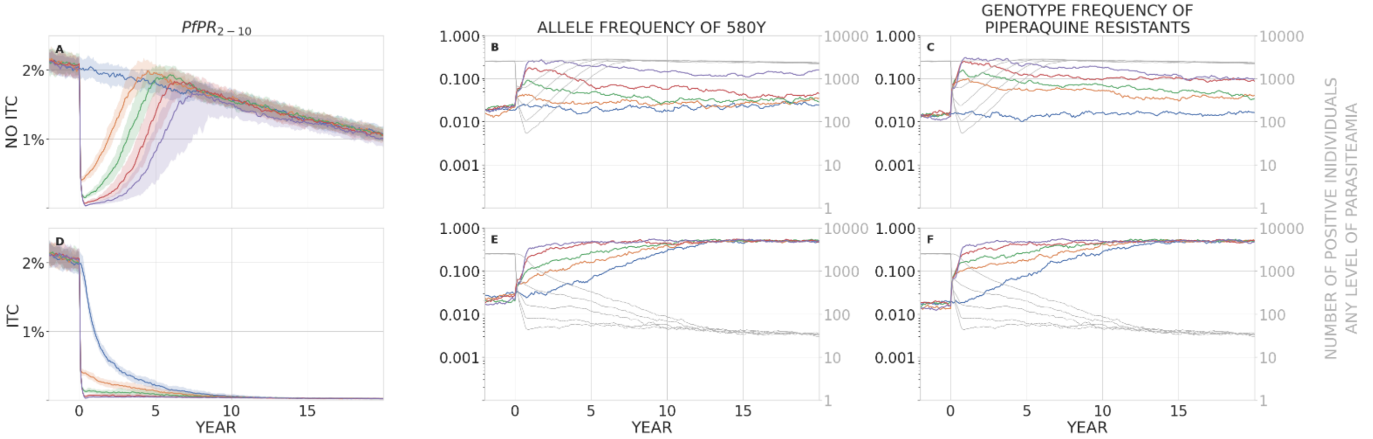

Fig C. AL in public sector was replaced by OZ439-FQ after the final round of MDA (for the blue line as well, on Jan 15 of year zero). AL is still used in the private sector. Note that in the bottom panels allele frequencies settle to 0.50 as the genotype frequencies are dominated by importation. In the absence of elimination (top panels) 580Y alleles gradually recede from the population.

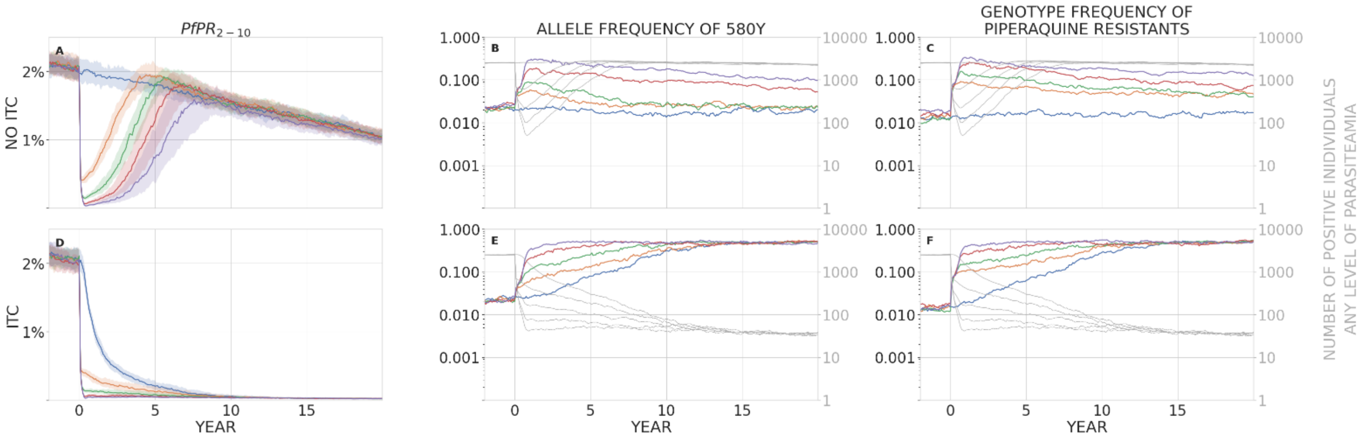

Fig D. Unlike in Fig C, AL is replaced by OZ439-FQ prior to the MDA. AL is still used in the private sector. Note that in the bottom panels allele frequencies settle to 0.50 as the genotype frequencies are dominated by importation. In the absence of elimination (top panels) 580Y alleles gradually recede from the population.

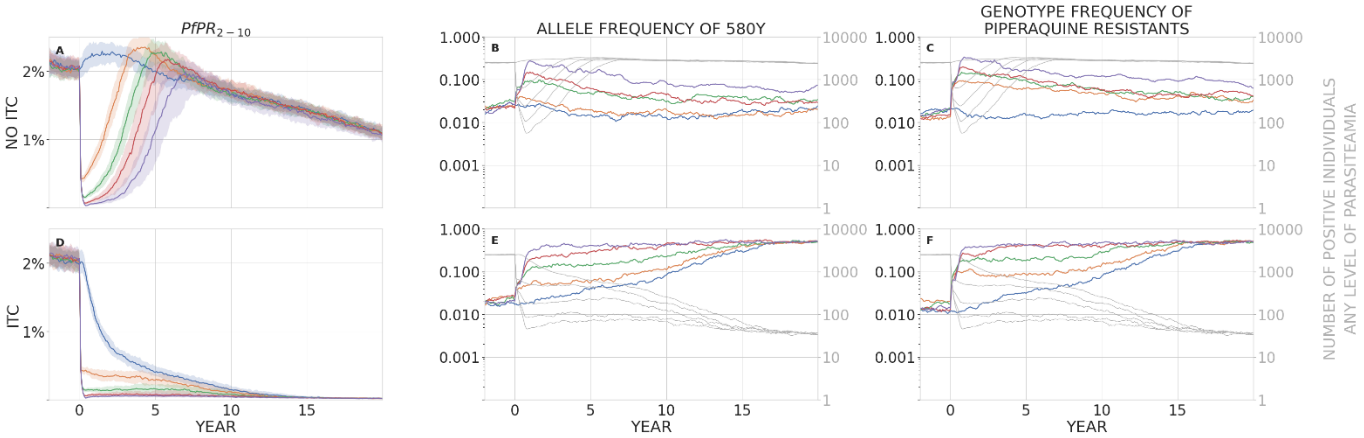

Fig E. AL is replaced by OZ439-FQ prior to the MDA, and AL is also removed from the private sector. 580Y frequencies in Panel B are lower when comparing to Fig D. Note that in the bottom panels, allele frequencies settle to 0.50 as the genotype frequencies are dominated by importation. In the absence of elimination (top panels) 580Y alleles gradually recede from the population.

4 Linkage Disequilibrium between 580Y and piperaquine resistance

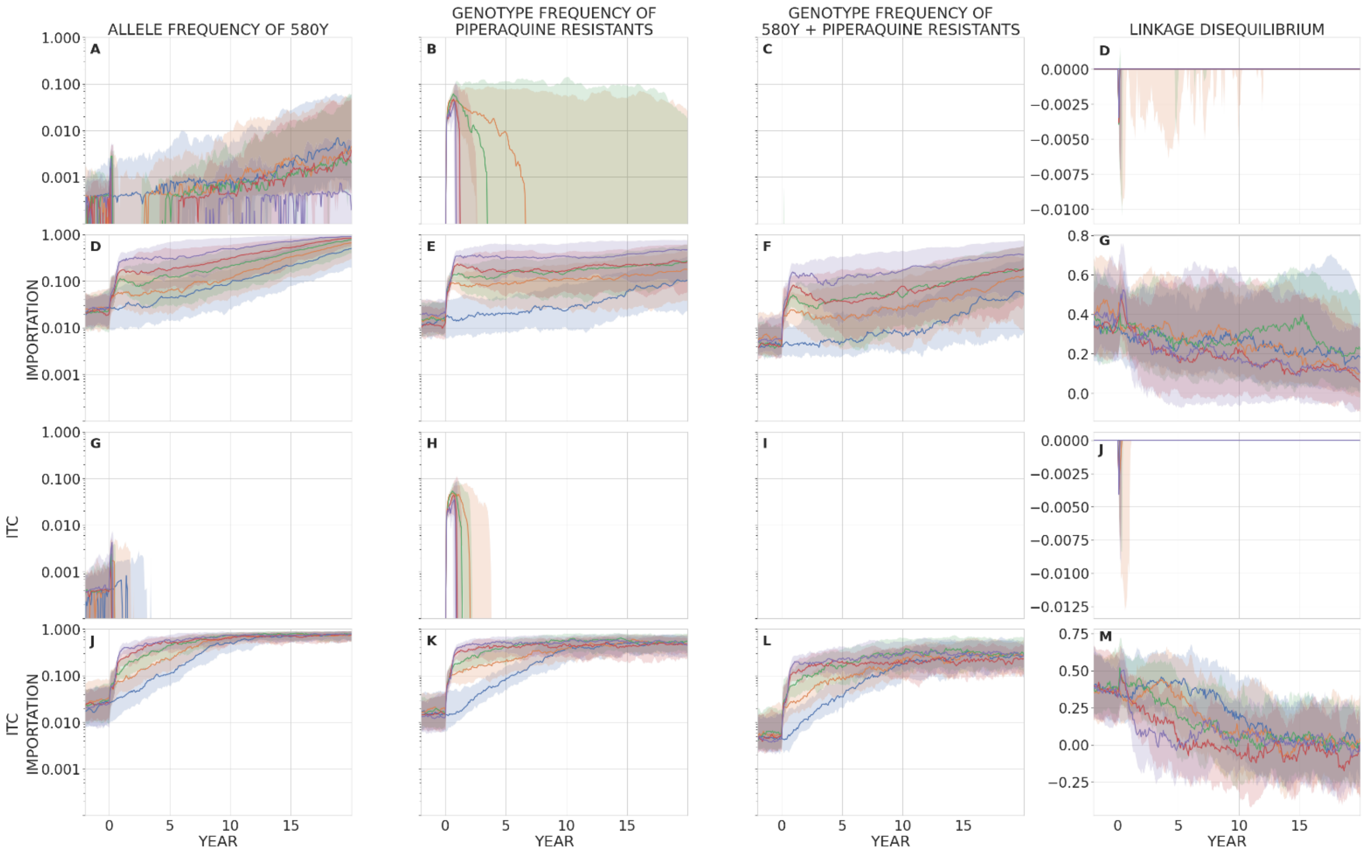

Fig F. Scenario settings are population size of 40,000 and 2% PfPR (as in Fig 2 of main text). Panels shows allele frequency of 580Y (first column), the frequency of piperaquine resistance (second column), the genotype frequency of 580Y plus piperaquine resistance (third column), and linkage disequilibrium (LD) between 580Y and PPQ-resistant genotypes (fourth column) over a period of 20 years after a mass drug administration has been carried out. In the second row LD is positive between the two genotypes (due to selection), and this helps maintain piperaquine resistance in the population even when PPQ is not used as part of the first-line therapy.

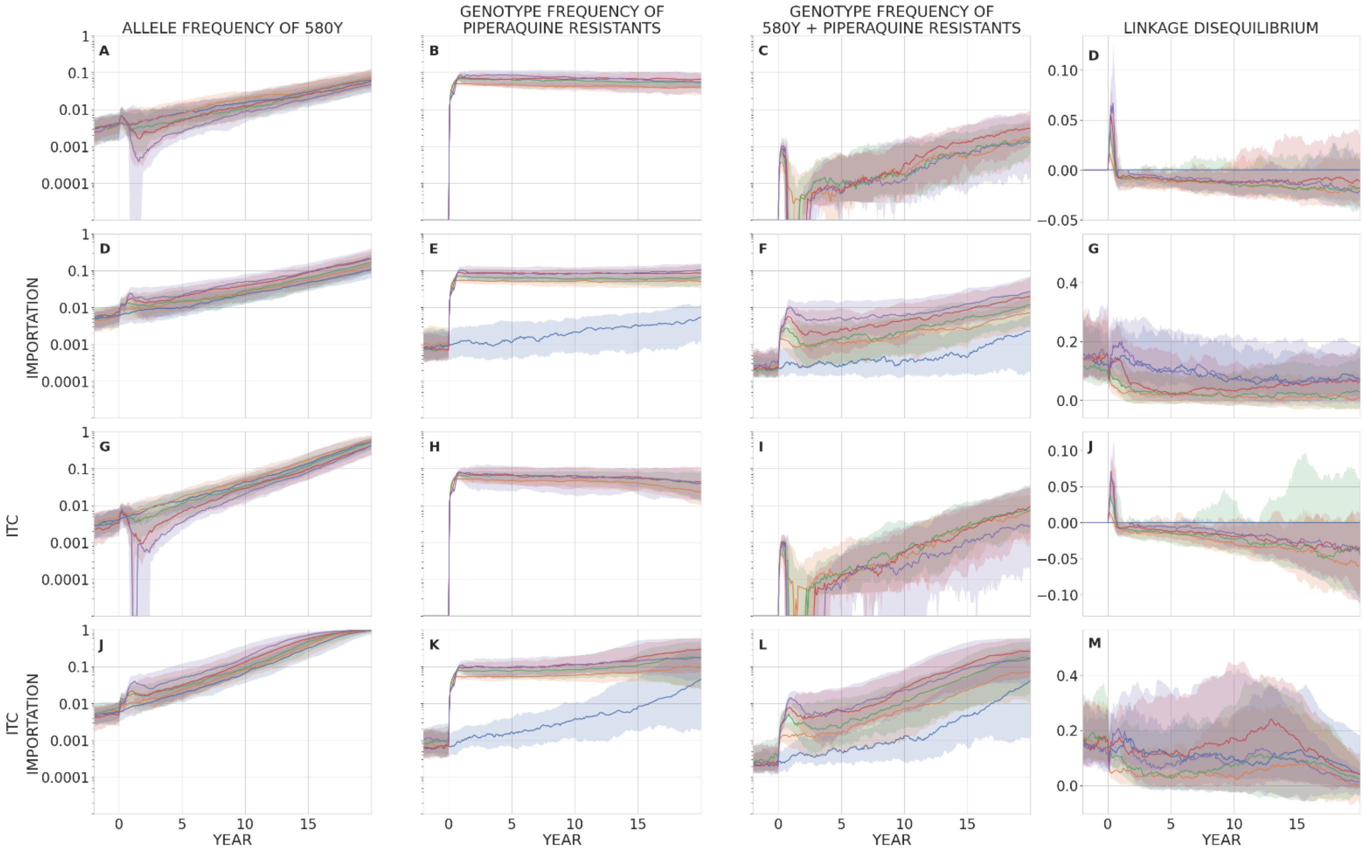

Fig G. Scenario settings are population size of 300,000 and 5% PFPR (as in Fig 3 of main text). Panels shows allele frequency of 580Y (first column), the frequency of piperaquine resistance (second column), the genotype frequency of 580Y plus piperaquine resistance (third column), and linkage disequilibrium (LD) between 580Y and PPQ-resistant genotypes (fourth column) over a period of 20 years after a mass drug administration has been carried out. In the second row and fourth rows LD is positive between the two genotypes (due to selection), and this helps maintain piperaquine resistance in the population even when PPQ is not used as part of the first-line therapy.

5 Sensitivity Analysis

To perform a sensitivity analysis on the factors influencing emergence of 580Y under different scenarios, Latin Hypercube Sampling (LHS, using *pyDOE* library in Python, https://pythonhosted.org/pyDOE/) was used to generate 50,000 data sets for the inputs and ranges as defined in Table A. As 'number of MDA rounds' cannot be sampled in an LHS format, this number was fixed between 0 and 4, and 10,000 parameter combinations were chosen via LHS for each number of MDA rounds. A total of 50,000 parameter combinations were in the final combined set. For each of the 50,000 simulations, the time until 580Y reaches an allele frequency of 0.25 ($T_{.25}$) was captured. The Partial Rank Correlation Coefficient (PRCC) between each parameter and $T_{.25}$ was calculated using the *epiR* package in R (https://cran.r-project.org/web/packages/epiR/index.html). All PRCC results are shown in Fig 6, main text.

Treatment coverage was not used as a parameter as this would affect the PfPR. Increasing treatment coverage would have the predicable effect of stronger selection pressure (lower $T_{.25}$) but this is not a property or result of the MDA program. MDA coverage was kept between 60% and 90%. MDA coverages below 60% are not worth considering as this would largely be viewed as an unsuccessful MDA program that did not have sufficient outreach.

**Parameter**	**Range**
Number of MDA rounds	[0 – 4], discrete only
Population Size in 2022	[40,000 – 300,000]
PfPR	[1% – 3%]
Importation rate	[0 – 0.2] cases per day
Improved Treatment Coverage (ITC) after MDA	[0.6 – 0.8]
Annual Cost of Resistance	[30% lower – 30% higher] compared to base line value
MDA Coverage	[0.6 – 0.9]

Table A. Parameters and ranges used in Latin Hypercube Sampling.

The Latin Hypercube Sampling also provides an opportunity to test the association between bottleneck size and 580Y allele frequency. The PRCC between the bottleneck size (smallest number of parasite-positive individuals at any time during the MDA) and the 580Y allele frequency was –0.31 in year 2 after the MDA, –0.27 in year 5, and –0.28 in year 8. All *p*-values from the PRCC package for these three associations are reported as zero. Essentially, as shown in Fig H, large bottlenecks are rarely associated with high 580Y frequencies. For larger bottlenecks, stochastic effects pushing low allele frequencies to high frequencies (purely by chance) do not occur very often.

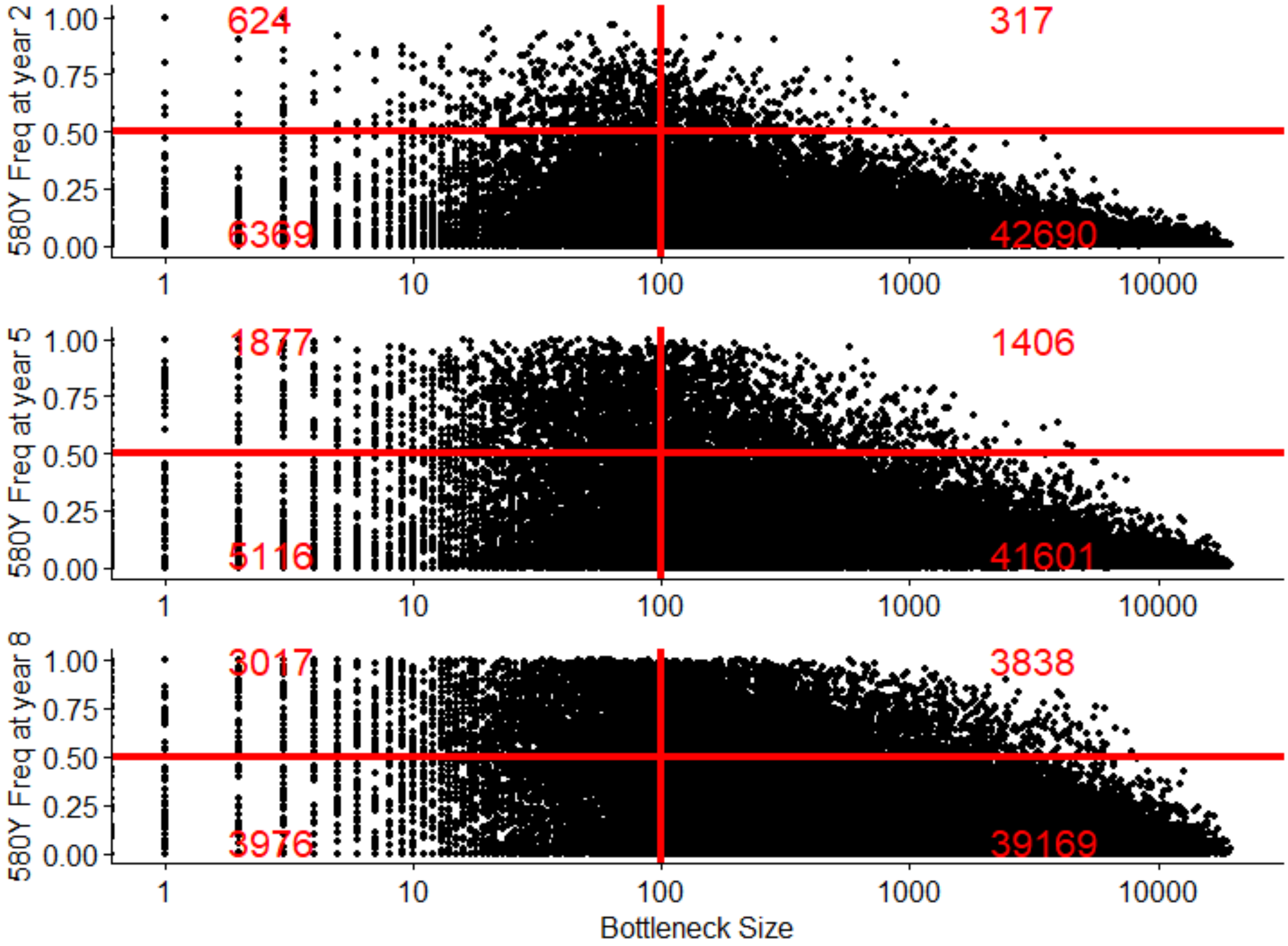

Fig H. Relationship between bottleneck size and 580Y frequency at years 2, 5, and 8 (frequency taken in January of each year). From the sensitivity analysis of 50,000 simulations, the bottleneck sizes (smallest number of parasite-positive individuals at any point during the MDA) and 580Y frequencies at various years were plotted. Each dot shows one simulation, and the red lines split the graphs into quadrants at a bottleneck of 100 individuals and a 580Y allele frequency of 0.50. The red numbers show the number of simulations in each quadrant. In general, 580Y frequencies do not evolve to high frequencies from large bottlenecks. Bottleneck size and 580Y frequency are negatively correlated in year 2 (Pearson $r = -0.2915$), in year 5 ($r = -0.3035$), and in year 8 ($r = -0.3261$). All p-values $< 2.2 \times 10^{-16}$.

6 Effects of varying efficacies of Sulfadoxine-Pyrimethamine (SP)

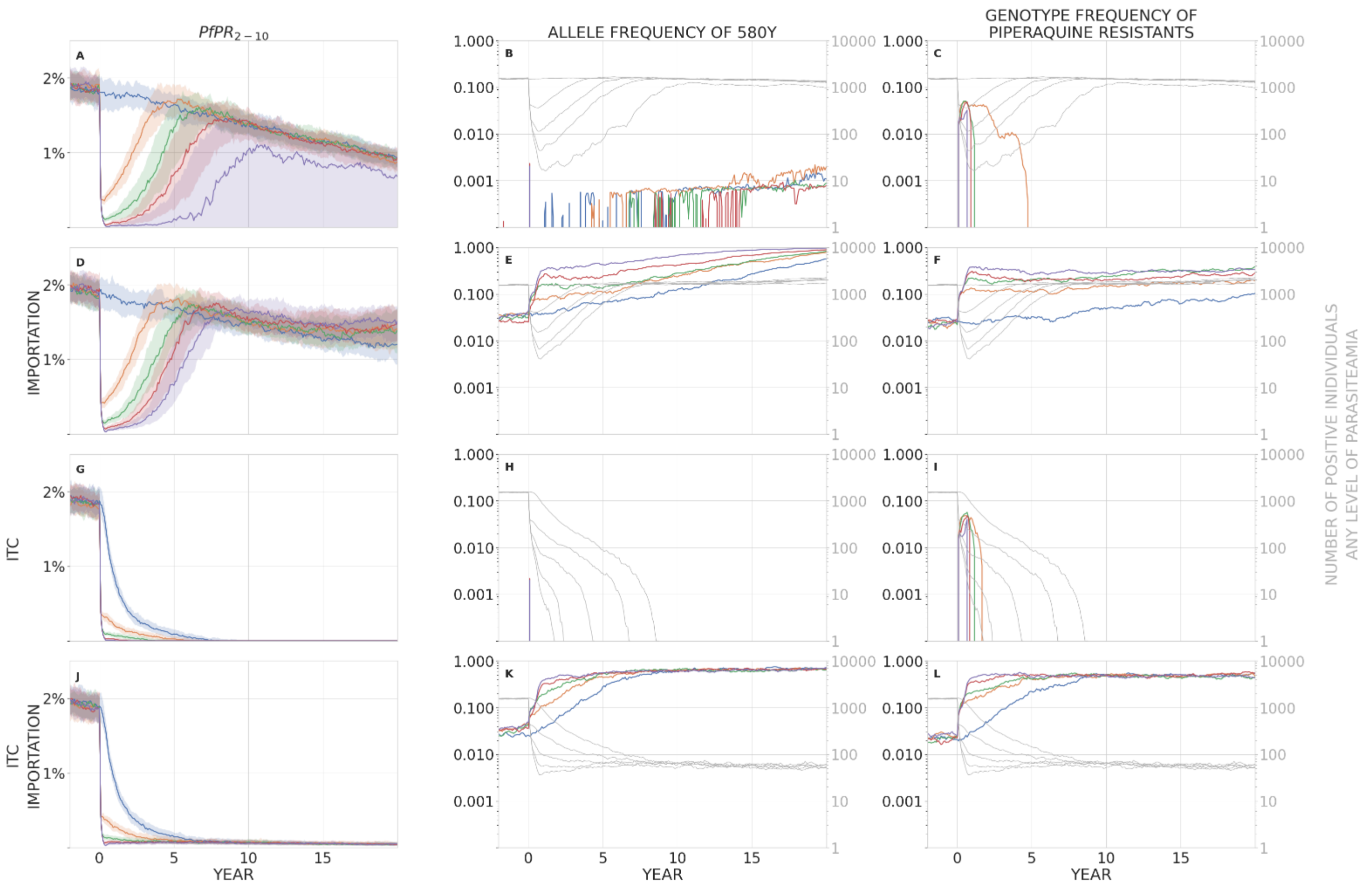

Fig I. Simulations run as in Fig 2 of the main text, but SP is assumed to have 60% efficacy across all genotypes.

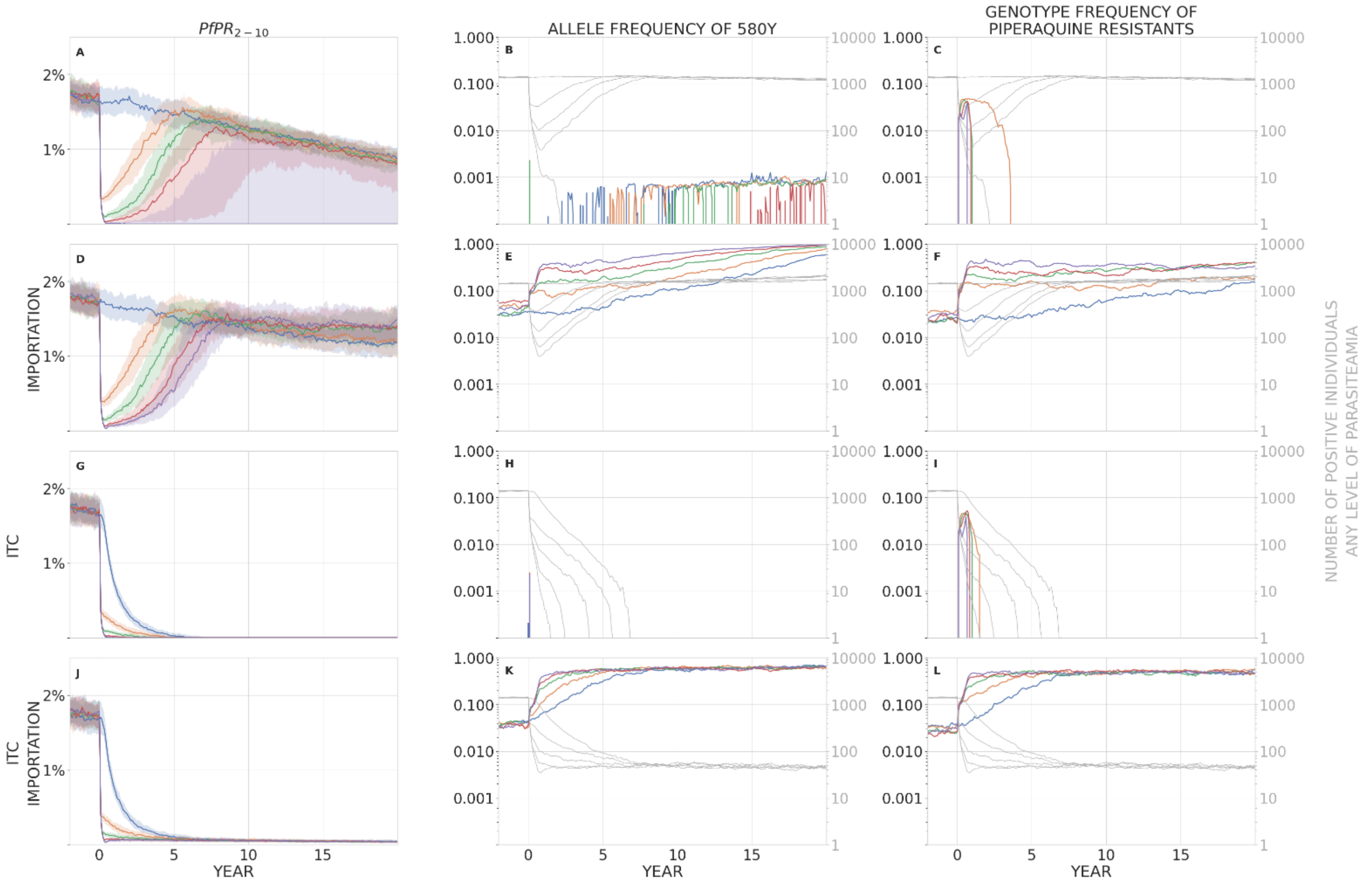

Fig J. Simulations run as in Fig 2 of the main text, but SP is assumed to have 80% efficacy across all genotypes.

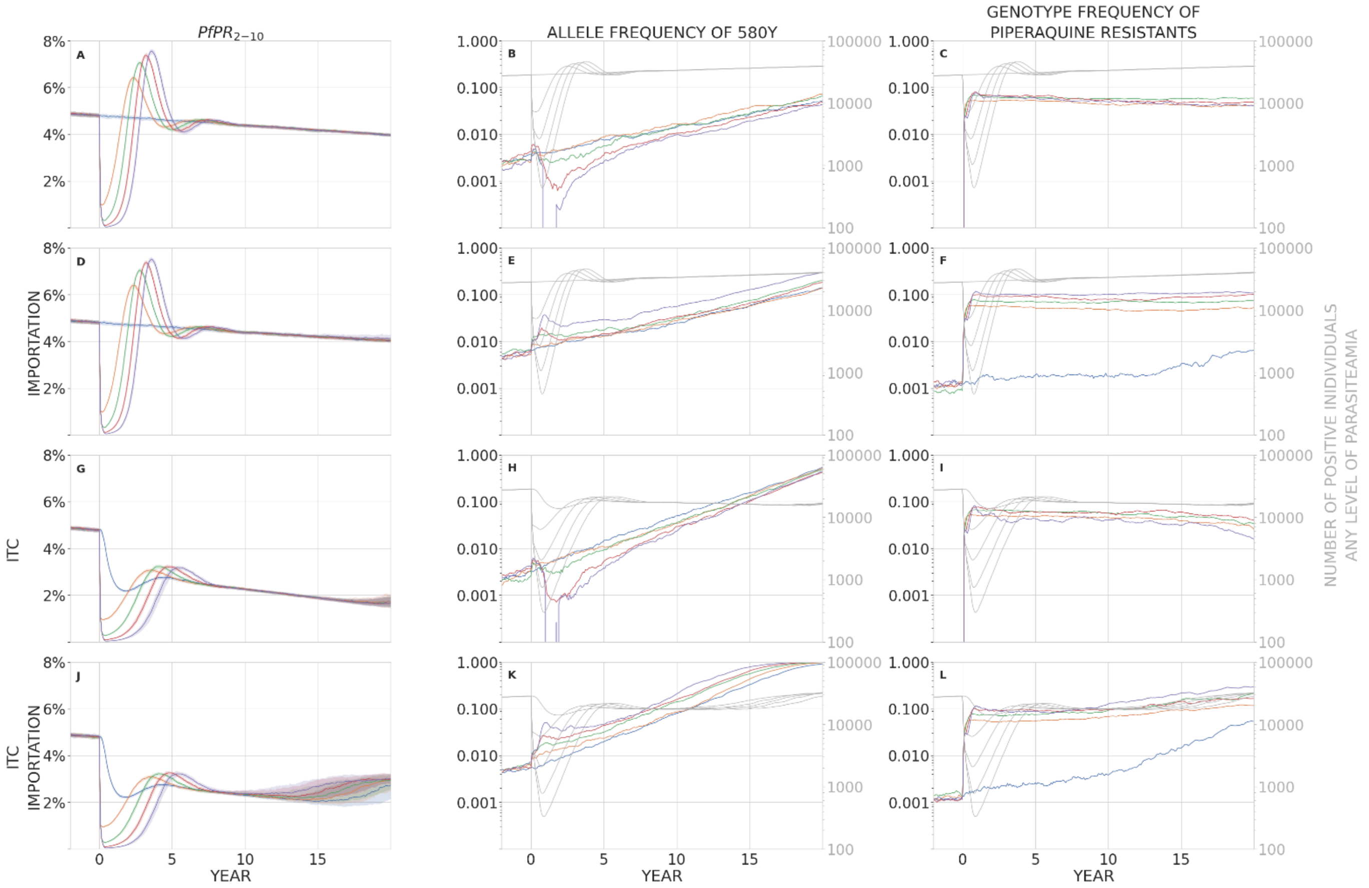

Fig K. Simulations run as in Fig 3 of the main text, but SP is assumed to have 60% efficacy across all genotypes.

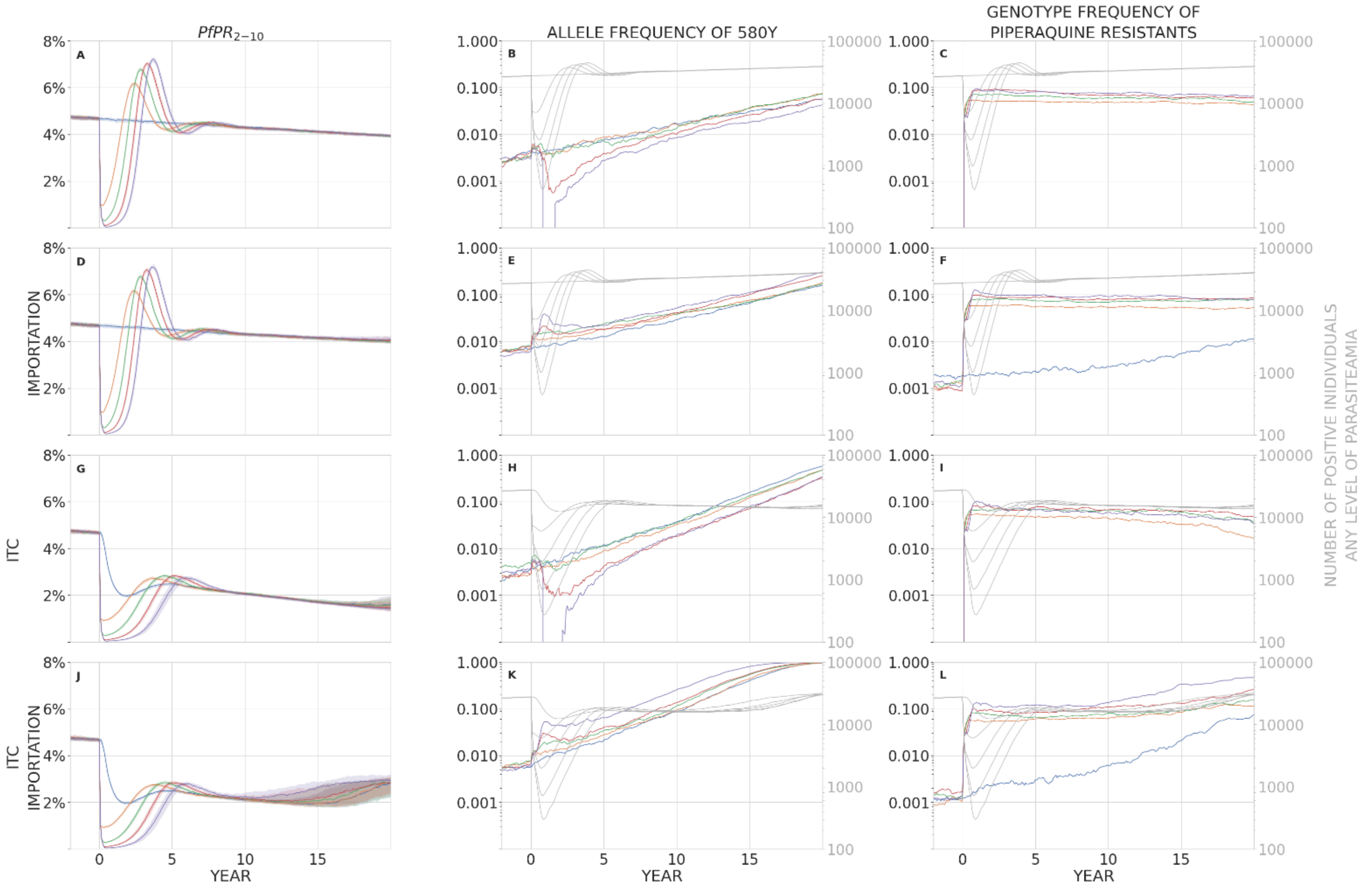

Fig L. Simulations run as in Fig 3 of the main text, but SP is assumed to have 80% efficacy across all genotypes.

7 Sensitivity to number of simulations performed

To determine if rare extinction, low migration, or other rare outcomes were affecting summary statistics we ran certain scenarios with both *N*=1000 and *N*=100 simulations.

Fig M and Fig N show the comparisons when simulating 40,000 individuals.

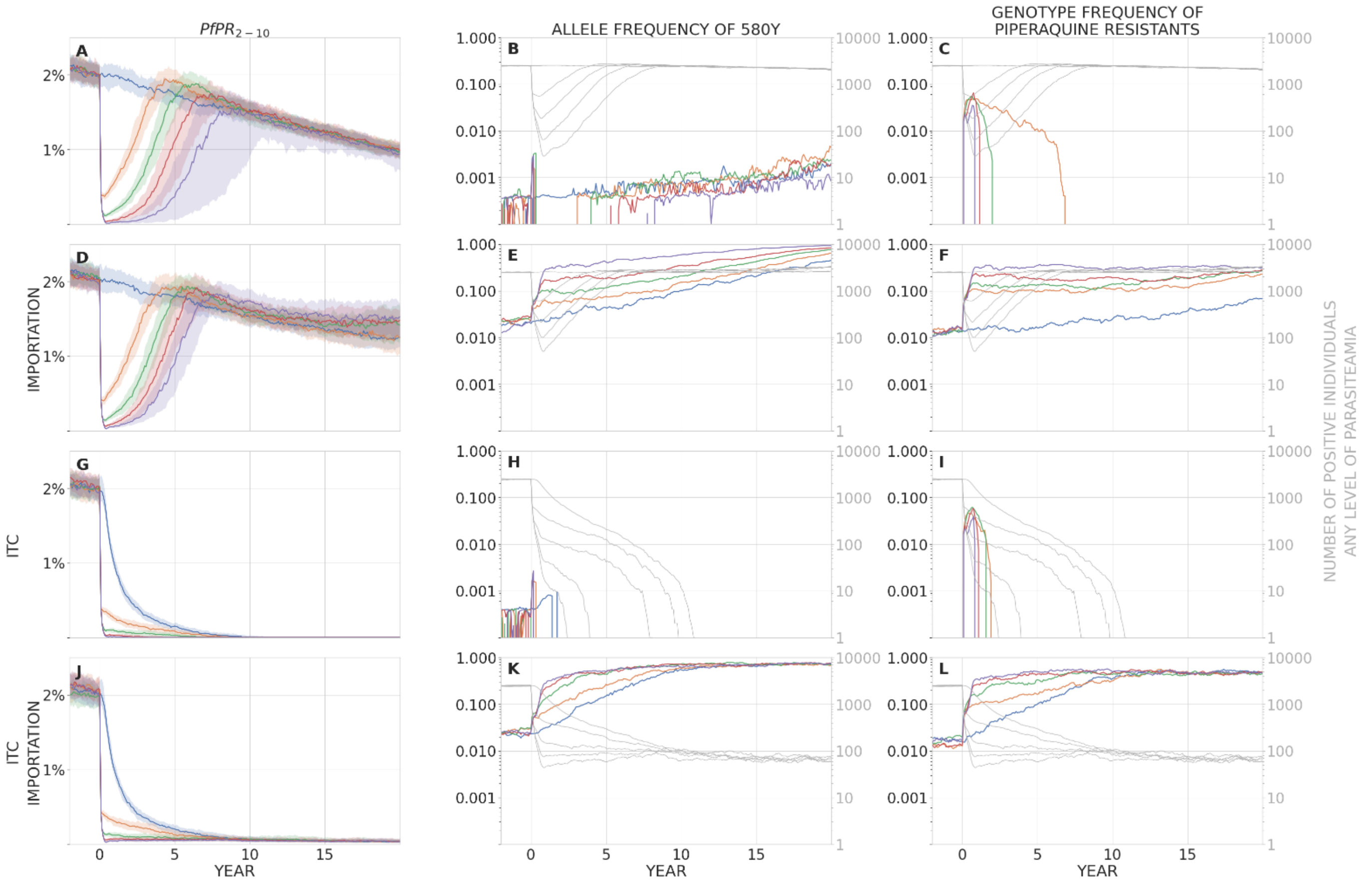

Fig M. This figure shows an exact copy of the scenario, parameterizations, and outputs of Fig 2 of the main text, but with *N* = 100 simulations. The malaria prevalence curves ($PfPR_{2\text{-}10}$, left column) are unchanged from *N*=100 to *N*=1000. With no importation, panels **B**, **C**, **H**, and **I** do not show any differences between N=100 and N=1000. With importation (panels **E**, **F**, **K**, **L**) the median genotype frequencies are lower for *N*=100 runs (above) than for *N*=1000 runs (Fig 2), suggesting that the stochasticity around the first introduction point of a successfully established 580Y mutant or piperaquine-resistant genotype does influence the future trajectory of drug-resistant genotypes, and that the distribution around this stochastic introduction may need to be modeled with a large number of simulations. Nevertheless, the relative evolutionary trajectories when comparing across the number of MDA rounds (0=blue, 1=orange, 2=green, 3=red, 4=purple) remains unchanged. The combination of (1) importation, (2) a small bottleneck, and (3) persistent selection pressure after the MDA still lead to higher higher long-term artemisinin-resistance under more rounds of MDA. The same conclusion is reached at 5% prevalence – compare Fig B to Fig N.

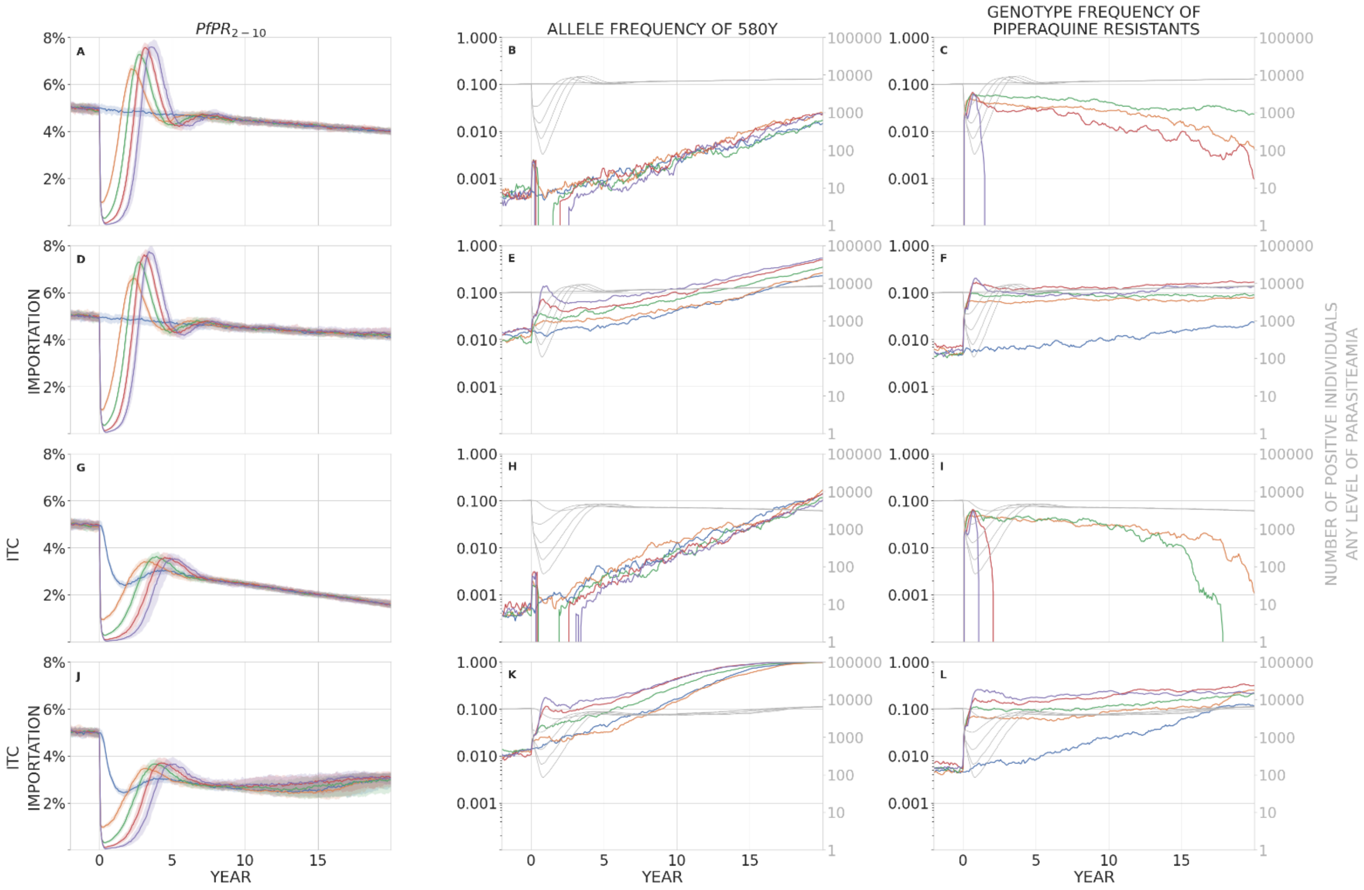

Fig N. This figure shows an exact copy of the scenario, parameterizations, and outputs of Fig B, but with $N = 100$ simulations. The median values in panels **E** and **K** do differ across N=100 and N=1000 simulations, but the ordering from zero rounds of MDA to four rounds of MDA does not.

Fig O and Fig P show the comparisons when simulating 300,000 individuals.

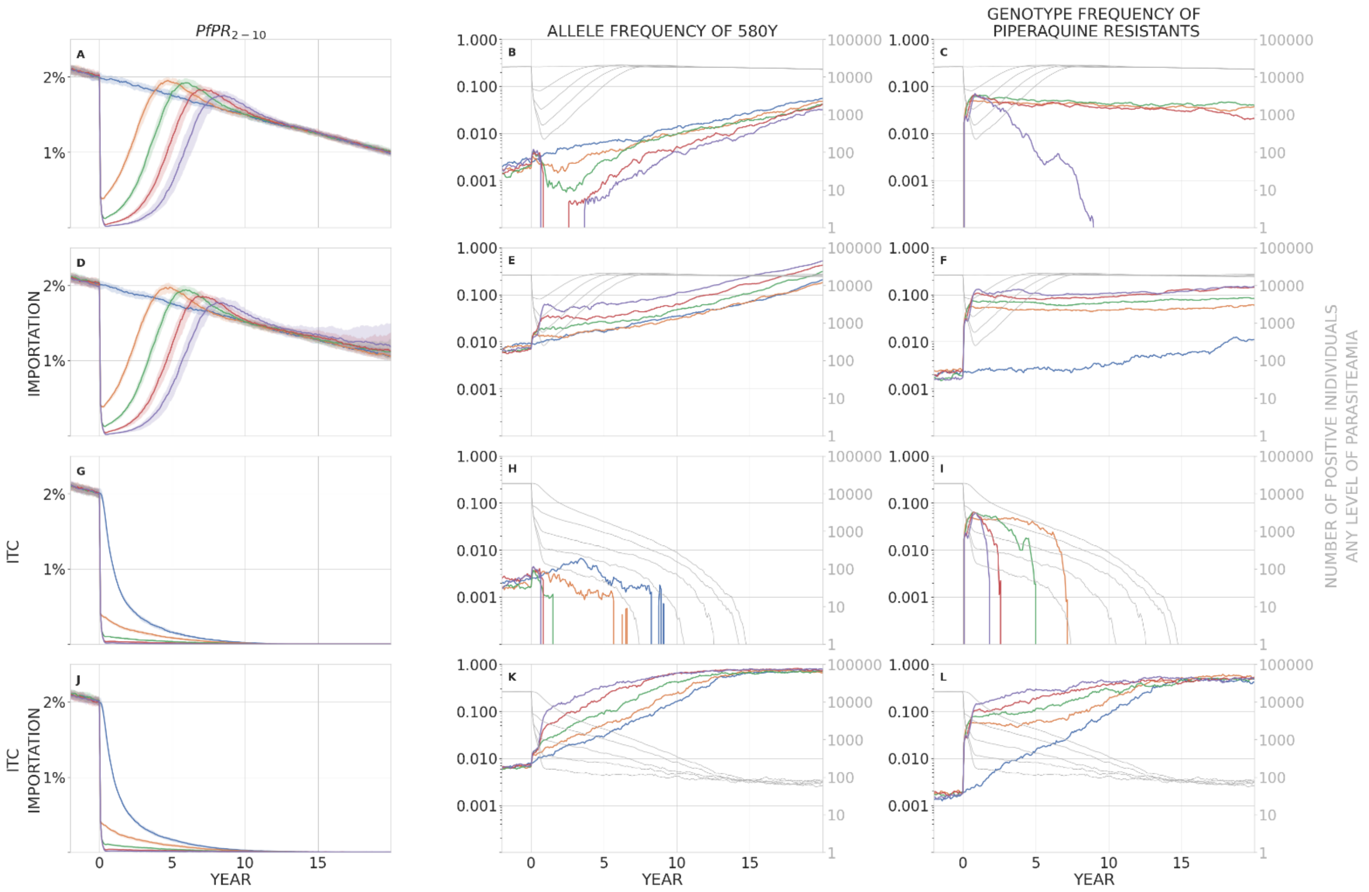

Fig O. This figure shows an exact copy of the scenario, parameterizations, and outputs of Fig A, but with $N = 100$ simulations. The median values in panels **E** and **K** do differ across N=100 and N=1000 simulations, but the ordering from zero rounds of MDA to four rounds of MDA does not.

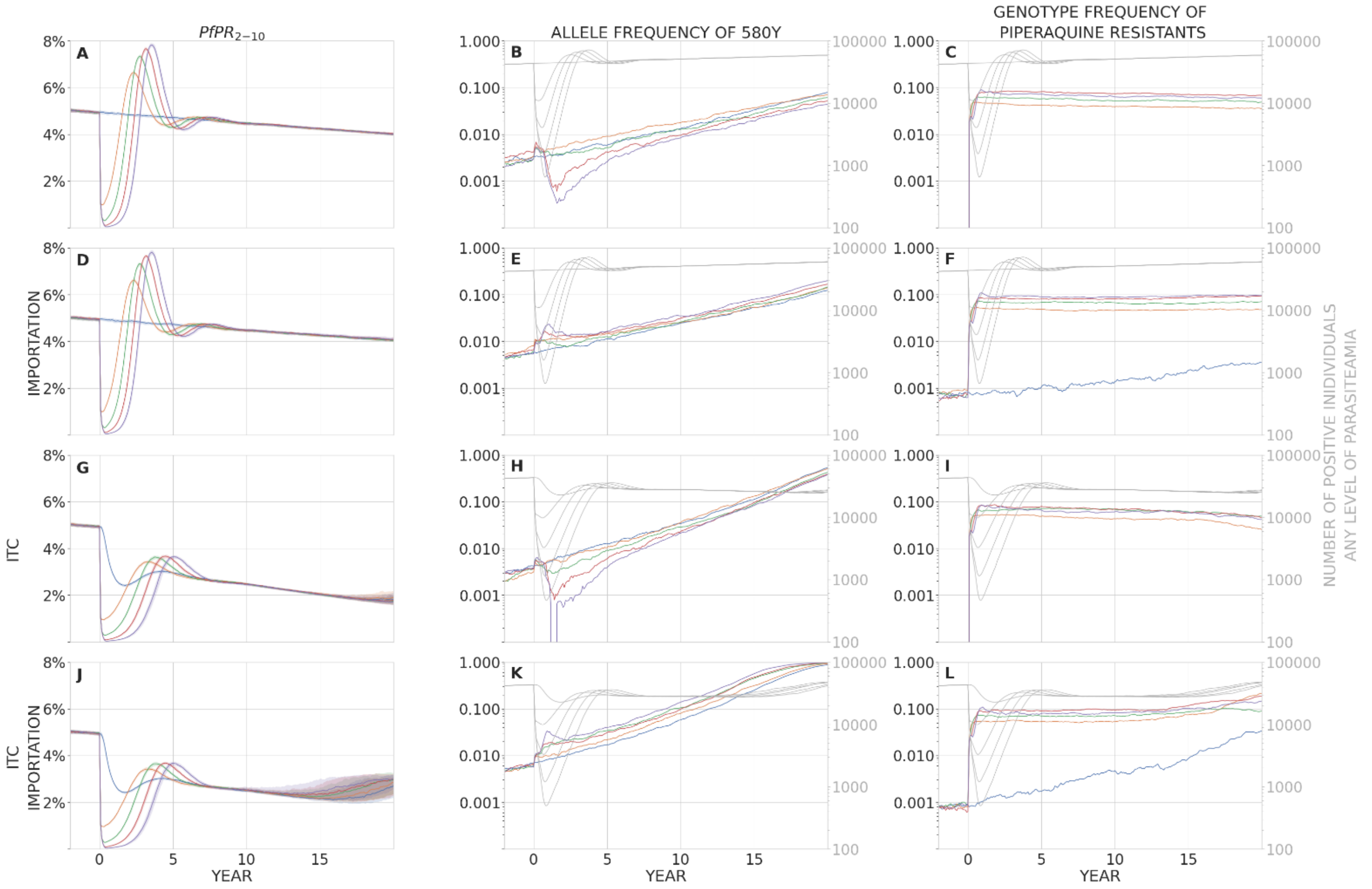

Fig P. This figure shows an exact copy of the scenario, parameterizations, and outputs of Fig 3 in the main text, but with N = 100 simulations. The median values in panels **E** and **K** do differ across N=100 and N=1000 simulations, but the ordering from zero rounds of MDA to four rounds of MDA does not.

8 Full range of variability for $N = 1000$ simulations

For *N*=1000 simulations, we show the 98% range of simulations in the following four figures.

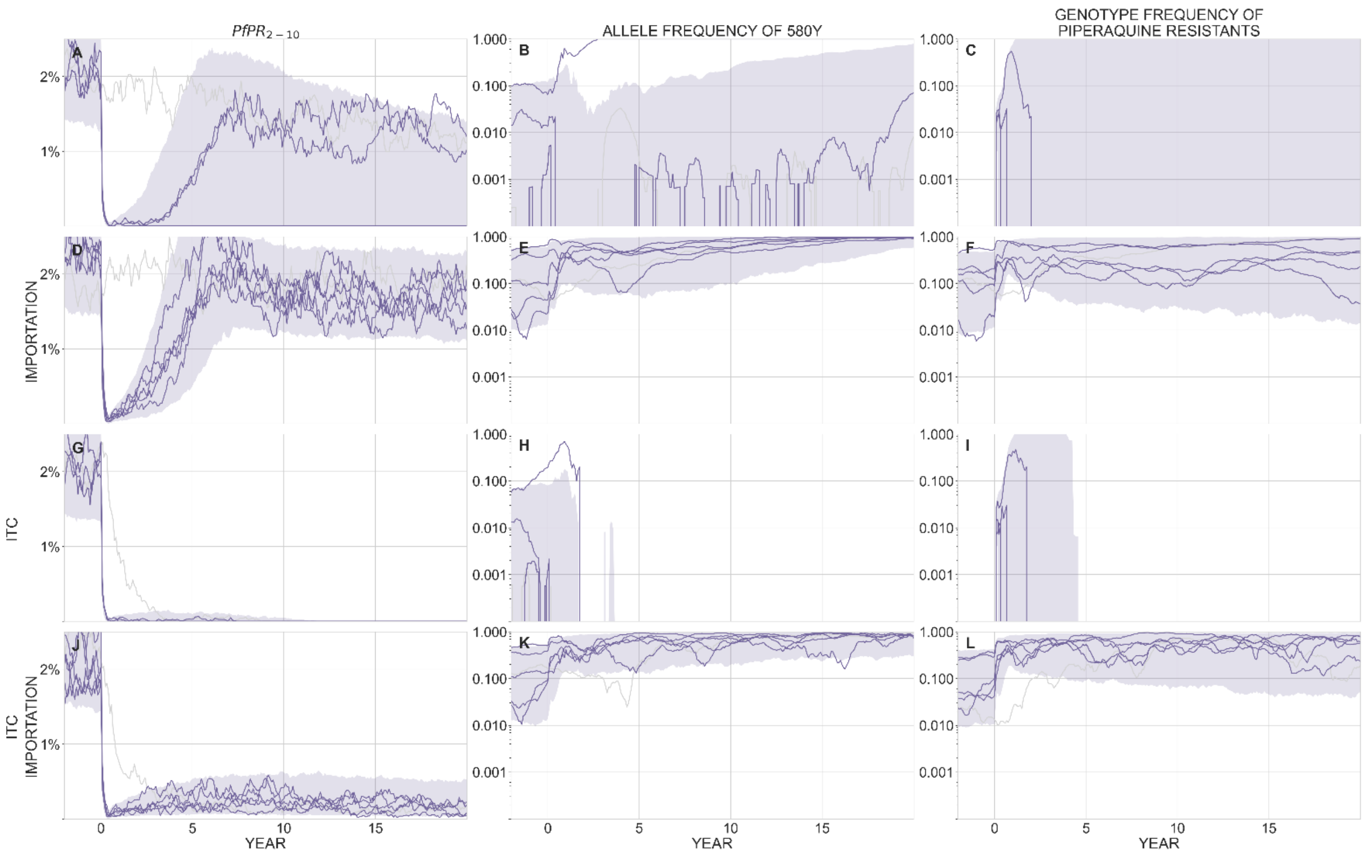

Fig Q. This figure uses the same simulation outputs as Fig 2 of the main text. The purple shaded area simply shows, for four rounds of MDA, the full range of outcomes from the 1st to the 99th percentile (*N*=1000 simulations, population size = 40,000 individuals). The five purple lines shown are individual simulation trajectories (and *not* composite medians of quartiles across *N*=1000 trajectorires). These five simulations were chosen by looking at the simulation output at six months post-MDA, ranking the 1000 simulations by the 580Y allele frequency at this point, and choosing the quantiles (.01, .05, .50, .95. .99) as the specific simulation runs to plot in all twelve panels. The gray line represents the median simulation for zero rounds of MDA (again, not a composite median, but the median simulation for 580Y frequency at six months post-MDA). In panels **H** and **I**, the allele frequency is predictable. In panels **D** and **J**, the prevalence pattern is predictable but the allele frequency is not. Only two simulations are shown in panel **A** because the other three reach extinction. Of these two, the 99th quantile simulation reaches fixation of 580Y after about 2.5 years, the exact perverse outcome we seek to avoid in MDA implementation.

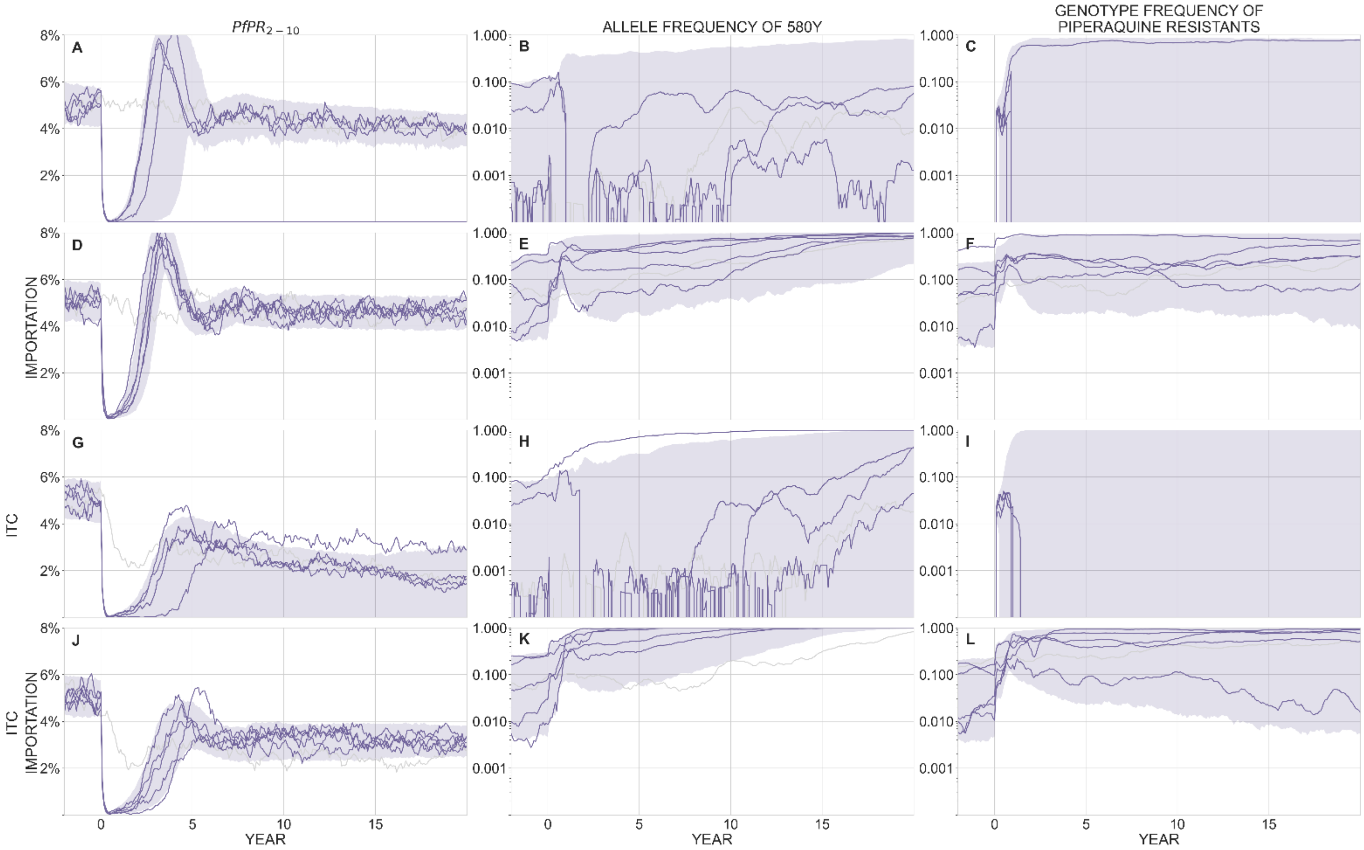

Fig R. This figure uses the same simulation outputs as Fig B. The purple shaded area simply shows, for four rounds of MDA, the full range of outcomes from the 1st to the 99th percentile (*N*=1000 simulations, population size = 40,000 individuals). Five purple lines and gray line chosen as in Fig Q. Allele frequency is not predictable for this $PfPR_{2\text{-}10}$ = 5% scenario; even in panel **K** the near-term 580Y allele frequency at five years ranges from 0.047 to 0.944. Prevalence outcomes are predictable in panels **A**, **D**, and **J**.

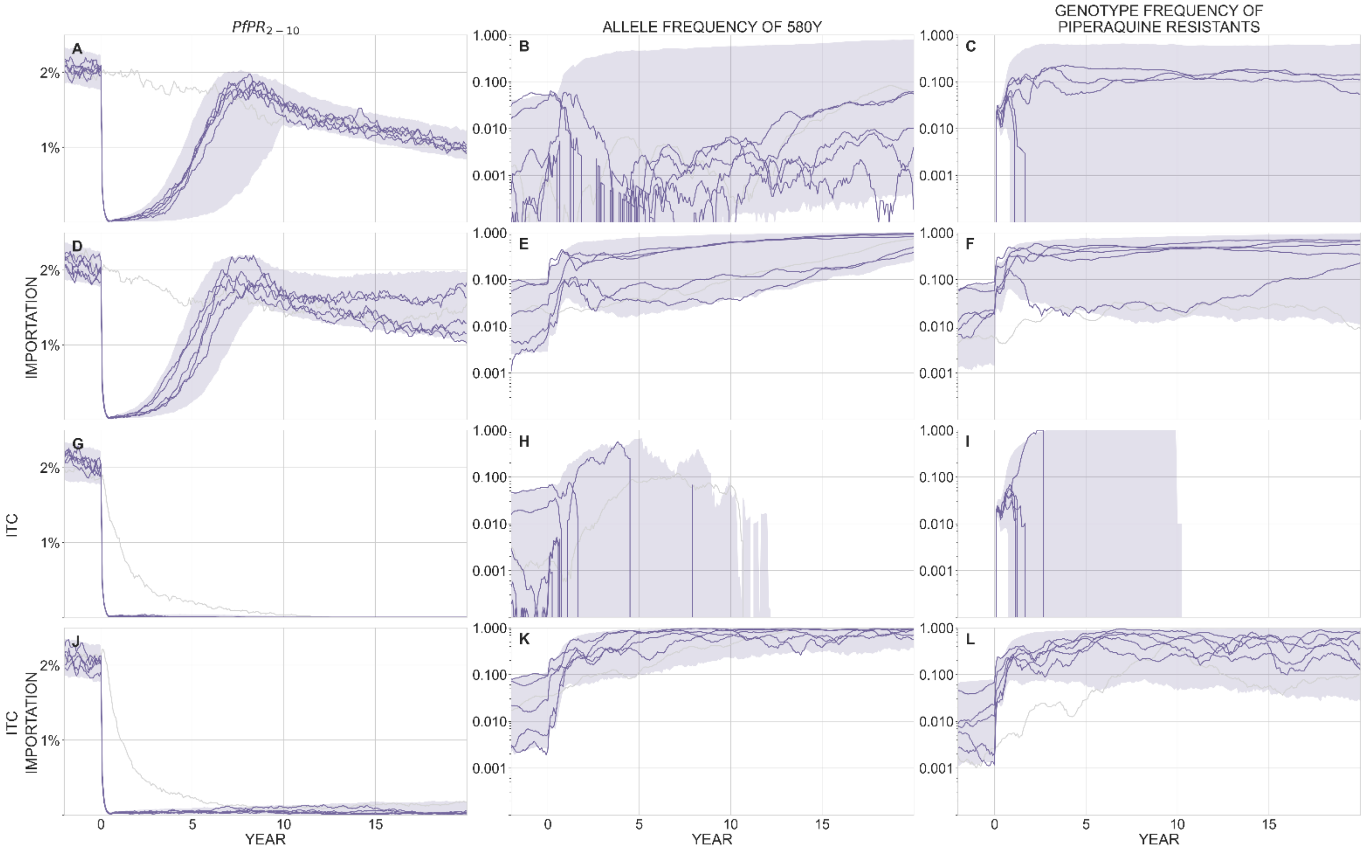

Fig S. This figure uses the same simulation outputs as Fig A. The purple shaded area simply shows, for four rounds of MDA, the full range of outcomes from the 1st to the 99th percentile (*N*=1000 simulations, population size = 300,000 individuals). Five purple lines and gray line chosen as in Fig Q. In panel **H**, the allele frequency is predictable; in 952/1000 simulations 580Y frequency stays below 0.20 for the entire simulation period. The prevalence pattern is predictable at $PfPR_{2\text{-}10}$ = 2%, 300,000 individuals, and four rounds of MDA.

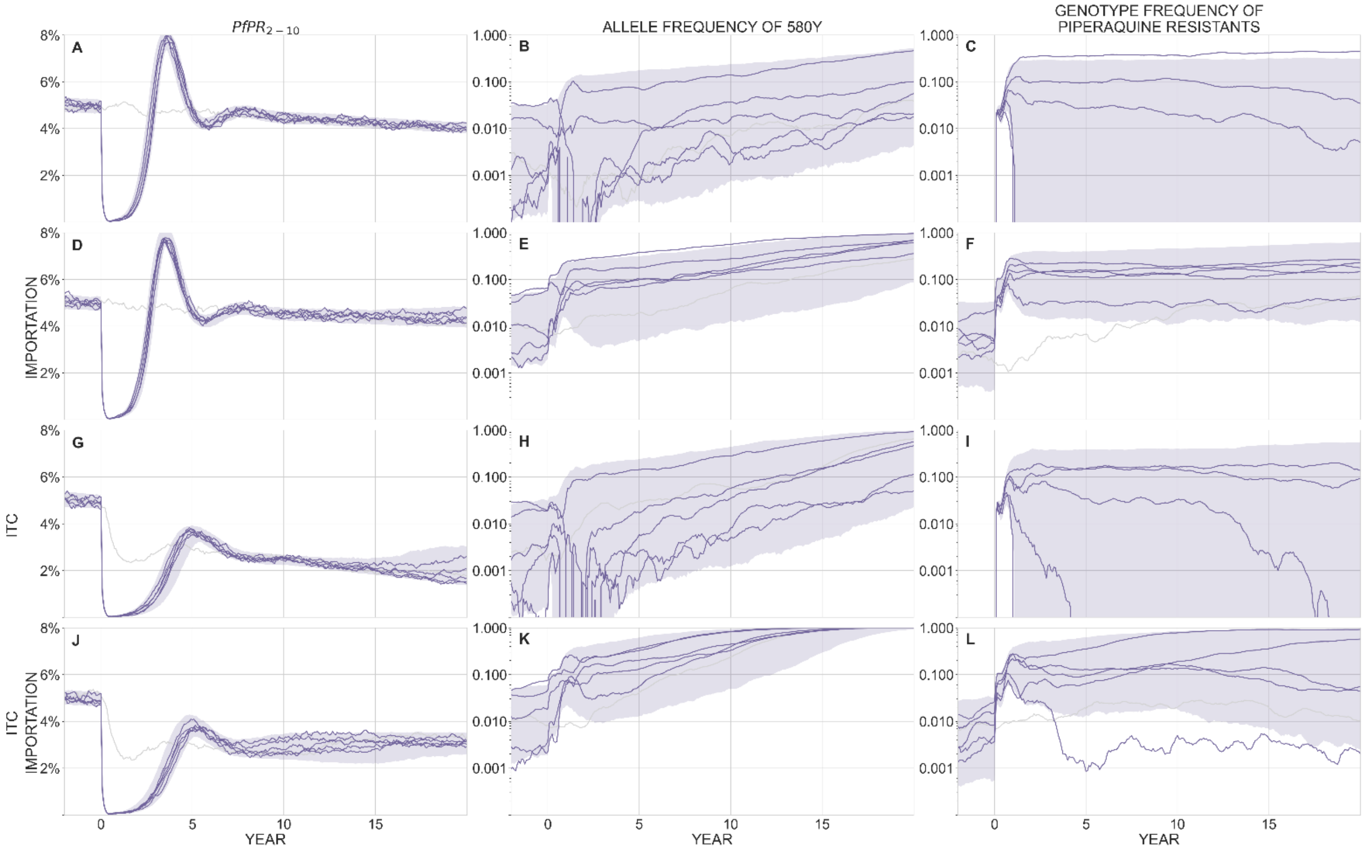

Fig T. This figure uses the same simulation outputs as Fig 3 of the main text. The purple shaded area simply shows, for four rounds of MDA, the full range of outcomes from the 1st to the 99th percentile (*N*=1000 simulations, population size = 300,000 individuals). Five purple lines and gray line chosen as in Fig Q. Allele frequencies are not predictable under this scenario. The prevalence pattern is predictable at $PfPR_{2\text{-}10}$ = 5%, 300,000 individuals, and four rounds of MDA.
