# Supplementary material for: Antimalarial mass drug administration in large populations and the evolution of drug resistance
Source: PLOS Glob Public Health. 2023 Jul 26;3(7):e0002200. doi: 10.1371/journal.pgph.0002200 (PMC10370688; doi:10.1371/journal.pgph.0002200)
Supplement: Attachment — Submitted filename: RefereeResponses.pdf [file pgph.0002200.s003.pdf]

Reviewer #1: This study raises a critical issue to consider when implementing MDA in different context. The manuscript is well written with comprehensive review of relevant evidences for constructing assumptions and model inputs. The conclusions and recommendations are important for public health intervention program to carefully plan to achieve the optimal outcomes to avoid future negative impact.

We thank the reviewer for these comments.

A minor suggestion: If the authors could provide few bullet points to summarize the most important findings, following by key recommendations related to MDA implementation, in a simple language that non-math people could understand, the readers will help accelerate the findings further, such as to policy makers.

This is an excellent suggestion. We have added a "Policy Box" with seven recommendations.

Reviewer #2: The goal of this study is timely for understanding the effect of MDA coverage on the emergence of antimalarial resistance. The proposed scenarios are well-designed and can lead to interesting conclusions, particularly regarding the time it takes for resistance to arise under specific conditions, such as importation of cases. However, a few issues require attention in the model, description of results, and discussion. In particular, a higher number of simulations would better support the results. The current number of 100 simulations for each combination of parameters is low, which may impact the validity of some of the results and curves in the figures. While the conclusions may not change dramatically, it is important to clearly indicate the limitations and more simulations are required.

This is an important comment. We redid our basic scenarios (40K or 300K individuals) for 2% and 5% prevalence – this is Figures 2, 3, S1, and S2. We replaced these figures with newer versions with results shown from 1000 runs instead of 100 runs. Figures S13 to S16 now have the old N=100 runs for comparison. We have added text to the methods describing this setup and some of the comparisons mentioned below.

First, the general results (i.e. everything in the policy box and abstract) are the same; our conclusions about the risk of drug resistance during MDA are unchanged.

Second, the referee is right that this matters. Drug resistance emergence and establishment are rare events, and Figures 2B, 2E, and 2K show clearly that at certain points in the simulation the total number of infected individuals drops down to the tens or hundreds. In addition, the timing of the first emergence and establishment event is a high-variance event, but this timing is what

determines the future trajectory of drug resistance and what the medians and quantiles of this process look like. Note for example that the medians are not the same across the panels. For example, in Figure 2 (N=1000) the median 580Y frequencies are higher than in Figure S13 (N=100), but the median piperaquine resistance frequencies tend to be higher at N=100 than at N=1000. These differences are small (this is stated in the S13 to S16 figure captions) but clearly there is some aspect of the stochasticity that is not being described in the summary figures. For this reason, we decided to plot a near-full range figures (1st to 99th quantile) for these four major figures, just to show how wide the variance was across simulations (with N=1000 runs), and these are now shown in Figures S17 to S20.

Third, a larger number of simulations allows you a clearer picture of the full range of simulation outcomes, and it allowed us to use Figures S17 to S20 to make an additional point about predictability, which was in our original ms but which we can now support with additional simulation and analysis. For example at 2% prevalence, Figures S17 and S19 show that allele frequency is only predictable in an "ITC-and-no-importation" scenario; otherwise the evolutionary path of artemisinin resistance spans nearly the entire range of allele frequencies from zero to one. This is now discussed in new section 3.3.

Fourth, we use Kruskal-Wallis tests to determine non-parametrically if there is any difference among the number of rounds of MDA applied. But, with N =1000 simulations, this test is very sensitive, and we routinely get back p-values in the 10^{-100} to 10^{-200} range. We have adjusted some of the descriptions of our statistics accordingly; all this new text is in green.

Finally, as the major conclusions (Policy Box, Abstract) are unchanged we did not repeat all analyses with N=1000 simulations per scenario. The analyses included in this paper comprise 330 simulated scenarios, and this would require 330,000 separate simulations to achieve an N=1000 sample set for each scenario. We did this for the four major scenarios outlined, but do not have sufficient computational capacity to run a total of 330,000 simulations.

I provide some specific (major and minor) comments on the manuscript as follows.

Abstract

The sentence in line 5 ("Effective MDA5… ") can be misleading since utilization of MDA might impose selective pressure. This is in fact acknowledged in the next sentences. I suggest revision.

This was perhaps just an awkward phrasing on our part, and it has now been revised.

Introduction

Line 40 - I recommend saying if genotypes are from P. falciparum, P. vivax or both. The Introduction does not make much distinction between these two species.

We have edited this. Our analysis is valid only for *P. falciparum*.

Model

Describe what EC50 is.

The EC50 is a metric used to quantify the concentration of a drug needed to achieve a response that is halfway between the baseline and the maximum daily rate of killing parasites during a specified period of exposure. In simpler terms, it represents the drug concentration at which the drug demonstrates 50% of its maximum effectiveness in reducing the daily parasite population.

We have added a short explanation.

Line 86: are these numbers arbitrary, such as 55% receiving treatment?

These are typical treatment coverage rates for Africa, based on the Bennett et al [31] measurements which are a bit lower and a bit older than current values. We could raise/lower the treatment coverage somewhat, but this would change the equilibrium prevalence in the model. So, we decided to simply adjust the prevalence and keep treatment coverage in the 55% to 60% range. Of course, for a particular specific regional scenario, we would redo all the simulations with as much local parameterization as possible.

Again, importation every 10 days seems arbitrary.

This is simply used as a "very high importation rate", i.e. a region where importation would be expected or routine. This is explained on lines 109-111. In Figure 4, we examine other importation rates, and it is in fact true (as expected) that the high or frequent importation scenario has the largest effect.

Regarding equal probability of importation of genotypes, does it not make sense that this importation would follow the frequencies of the most common sources of importation?

Yes, that's right. Since we are not modelling any specific country scenario here, we did not seek out the data on neighboring country/region genotype frequencies. The equal probability choice

was made to ensure that every genotype could be equally selected when imported (e.g. no differential effects in random stochastic die-offs across genotypes).

Line 145 (section 2.3): this seems to be results reported in the methodology? If so, I suggest to have it in the next section.

We have rephrased this. This is more the methodology of our calibration and scenario selection. We are not intending to communicate results on high-prevalence settings, we are simply explaining how we chose the scenarios we modeled.

Line 160 - 174: part of this material could appear in Introduction or Discussion.

We realize this is unusual, but we wanted the first paragraph of our results section to be an easy to understand ‘results summary’, to give the reader the big picture on the evolutionary forces acting in this system. Very little is published on the evolutionary outcomes of malaria policy, and we would like the results to be understood as clearly as possible.

Of course we could do this via Figure 6 or at the beginning of the Discussion, but we thought it would be easier for the reader to have a general description of the results first (i.e. what forces drive drug resistance evolution during MDA), and then get familiar with each specific scenario afterwards. We made Figure 1 specifically for this reason, as a pedagogical tool, to walk the reader through a general description of the relevant evolutionary forces.

If the editor or referee strongly disagree with this placement, we can lead the discussion with this paragraph.

Description of T0.25 is required.

Yes, thank you, we have clarified this at the end of section 2.2.

How is the Fitness cost modeled?

Apologies, this was our omission in the original manuscript. A few sentences (in green) are now included in section 2.1

How many simulations are run in each scenario?

1000 simulations for the four main scenarios (Figs 2, 3, S1, S2), and 100 for all others. This is now clear in the text.

Results

Line 192: elimination observed for three simulations (8/100), anything misreported here?

This has been updated due to the denominator changing from 100 to 1000. The new text here is correct.

Line 266 - 275: sounds as Introduction or Discussion

Yes, true. This is here to explain the rationale for the next two paragraphs, so the readers see why we carried out the ITC analysis. We think this is the best place for this paragraph, but again, if the editor/referee feel strongly, we can move this paragraph.

Line 295 - 299: This seems more a material for the Discussion

Yes, thank you, and this is already in the Discussion. Two sentences were removed here.

Lines 330 - 332: critical for discussion

Yes, thank you, and this is re-visited in the discussion and in the new Policy Box under Recommendation 4.

Figure 1: indicate the meaning of orange box and other boxes and colors. Need to find a more meaningful label than Num Pos Indivs. What scale is for the x-axis?

Label and x-axis description have been updated, thank you. The colors of the boxes correspond to the interquartile regions in the top right panel (this is stated at the end of the figure caption).

For instance, in Figure 1 I expect that when plotting the average numbers the curves will be better behaved. The same is likely to happen to Figure 2 in the scenarios without importation.

Figure 1 is just a schematic, showing that elimination generally occurs in small populations, and that the genotype distribution is pushed into a “resistance bottleneck” as this is happening. The quantiles themselves will be better behaved with a larger number of simulations, but in real life a particular trajectory or realization will not be well behaved. The current figure reflects this.

The average numbers aren’t shown because we have chosen medians and quantiles as our main statistics of interest that we are displaying.

For Figure 2, these simulation runs were indeed updated to $N = 1000$ based on the referee’s recommendation and the quantile curves are in fact better behaved.

Figure 3: for panels F and L, one of the curves is much lower. What is the explanation?

These are the PPQ-resistance curves, and the blue lines show the curves when no MDA was carried out (i.e. no DHA-PPQ was used and there was no selection pressure for PPQ-resistance). All of the other curves had selection pressure, during the MDA, for PPQ-resistance.

The blue line, just shows the level of PPQ-resistant genotypes under (1) importation and (2) linkage to 580Y genotypes which are selected for by artemether-lumefantrine use. There are additional graphs and explanations under Figure S7.

Figure 4: Is this figure indicating that for low levels of importation it will take 20 years to reach 25% of resistance? Since the maximum time in the simulations was 20 years, then it seems that 20 years appears here by an artifact of this simulation scenarios.

Yes, that’s right, this just means “20 or more years”. The figure caption has been edited.

I would recommend highlighting the effects, in particular the negative effect in the sensitivity analysis for the importation rate and the MDA coverage.

(*) Yes, fully agree, these are marked in green in the Results and at the end of section 4.1. Our word count is already quite high. If there is any other specific place in the text where these results should be highlighted, we can consider adding this in.

Discussion

What do the authors mean with “drive parasite numbers to zero while the number of parasite-carrying individuals is still low”?

This just means “use some policy to reduce total number of infections to zero” so that malaria is locally eliminated and will not rebound unless there are reintroductions.

It is easiest to drive “number of infections” to zero when the current number of infections is low. This is the pre-elimination phase according to WHO terminology.

The sensitivity analysis deserves a discussion especially in terms of the negative effects that were found.

Yes, this has been done, please see point (*) above.
